# *In Vivo* Control of CpG and Non-CpG DNA Methylation by DNA Methyltransferases

**DOI:** 10.1371/journal.pgen.1002750

**Published:** 2012-06-28

**Authors:** Julia Arand, David Spieler, Tommy Karius, Miguel R. Branco, Daniela Meilinger, Alexander Meissner, Thomas Jenuwein, Guoliang Xu, Heinrich Leonhardt, Verena Wolf, Jörn Walter

**Affiliations:** 1 Department of Biological Sciences, Institute of Genetics/Epigenetics, University of Saarland, Saarbrücken, Germany; 2 Department of Computer Science, University of Saarland, Saarbrücken, Germany; 3 Epigenetics Programme, Babraham Institute, Cambridge, United Kingdom; 4 Centre for Trophoblast Research, University of Cambridge, Cambridge, United Kingdom; 5 Department of Biology II, LMU München, Biozentrum Martinsried, Martinsried, Germany; 6 Department of Stem Cell and Regenerative Biology, Harvard University, Cambridge, Massachusetts, United States of America; 7 MPI for Immunology and Epigenetics, Freiburg, Germany; 8 Institute of Biochemistry and Cell Biology, Chinese Academy of Sciences, Shanghai, China; Friedrich Miescher Institute for Biomedical Research, Switzerland

## Abstract

The enzymatic control of the setting and maintenance of symmetric and non-symmetric DNA methylation patterns in a particular genome context is not well understood. Here, we describe a comprehensive analysis of DNA methylation patterns generated by high resolution sequencing of hairpin-bisulfite amplicons of selected single copy genes and repetitive elements (LINE1, B1, IAP-LTR-retrotransposons, and major satellites). The analysis unambiguously identifies a substantial amount of regional incomplete methylation maintenance, i.e. hemimethylated CpG positions, with variant degrees among cell types. Moreover, non-CpG cytosine methylation is confined to ESCs and exclusively catalysed by Dnmt3a and Dnmt3b. This sequence position–, cell type–, and region-dependent non-CpG methylation is strongly linked to neighboring CpG methylation and requires the presence of Dnmt3L. The generation of a comprehensive data set of 146,000 CpG dyads was used to apply and develop parameter estimated hidden Markov models (HMM) to calculate the relative contribution of DNA methyltransferases (Dnmts) for *de novo* and maintenance DNA methylation. The comparative modelling included wild-type ESCs and mutant ESCs deficient for Dnmt1, Dnmt3a, Dnmt3b, or Dnmt3a/3b, respectively. The HMM analysis identifies a considerable *de novo* methylation activity for Dnmt1 at certain repetitive elements and single copy sequences. Dnmt3a and Dnmt3b contribute *de novo* function. However, both enzymes are also essential to maintain symmetrical CpG methylation at distinct repetitive and single copy sequences in ESCs.

## Author Summary

DNA methylation is a stable covalent epigenetic modification of cytosines mostly confined to CpG-dinucleotides in mammals. In general, it is associated with silencing of genomic DNA regions. Three catalytically active DNA methyltransferases (Dnmts) set and maintain CpG methylation in cooperation with other (co-)factors. The *in vivo* contribution of the Dnmts to maintain CpG and non-CpG methylation following rounds of DNA replication are not well understood, particularly since *in vivo* DNA methylation patterns can be highly dynamic. In our work, we use ultradeep sequencing to determine the methylation status of both DNA strands in ESCs depleted for Dnmts 1, 3a, 3b, and 3L, respectively. Using hidden Markov models, we calculate the relative contribution of each of the enzymes for the maintenance of DNA methylation patterns using parameter estimated fitting. While in general the modelling supports a classification of Dnmts into maintenance and *de novo* functions, it argues against a strict enzyme specific functional categorisation. We observe evidence for a context-dependent contribution of Dnmts to set and maintain CpG and non-CpG methylation at distinct classes of repetitive elements and selected single copy genes. We furthermore unambiguously identify Dnmt3a/3b and 3L dependent non-CpG methylation at specific sequence positions and confined to ESCs.

## Introduction

DNA methylation at the C-5 positions of cytosine (5mC) is a key epigenetic modification in mammals essential for normal development [1], [2]. Cytosine methylation is predominantly found in CpG dinucleotide context and about 70 to 80% of all CpGs are methylated. These methylated CpGs are usually located in CpG poor regions and often in repetitive sequences [3]–[5]. About 40% of the genome consists of repetitive elements. Four main groups of repetitive elements can be discriminated: long interspersed nuclear elements (LINEs), short interspersed nuclear elements (SINEs), long-terminal repeat (LTR) retrotransposons and (peri-) centromeric satellites. For these elements, the maintenance of DNA methylation during development and aging is important for transcriptional silencing and genome stability [6], [7].

The establishment and maintenance of methylation patterns at palindromic CpG sequences (CpG dyads) is performed by three catalytically active DNA methyltransferases (Dnmts). *In vitro* experiments suggest that Dnmt1 prefers hemimethylated CpG (hemi-mCpG) dyads and maintains the methylation pattern on the newly synthesized strand after replication (maintenance methylation). In *vitro*, Dnmt1 shows a low activity on unmethylated CpG dyads. Dnmt3a and Dnmt3b methylate DNA *de novo*, independent of the methylation status of the complementary CpG position [8], [9]. I*n vitro* analyses furthermore suggest that methylation by Dnmt3b and the maintenance function of Dnmt1 mostly occur in a processive manner, whereas Dnmt3a and the “*de novo* function” of Dnmt1 are distributive [9]–[12]. However, other groups observe a processive methylation activity for Dnmt3a [13]. In addition, Dnmt activities are modulated by Nuclear protein of 95 kDa (Np95; also known as Uhrf1) and Dnmt3L. Dnmt3L, a cofactor for the *de novo* methyltransferases, is reported to stimulate Dnmt3a/3b activity, to be needed for *de novo* establishment for imprint methylation and furthermore to enhance the processive methylation activity of human Dnmt3a [13]–[15]. Np95 is recruiting Dnmt1 to hemimethylated DNA and is interacting with Dnmt3a/3b for gene silencing [16]–[18].

Despite of a lot of *in vitro* data on Dnmt specificities and interacting partners, relatively little is known about the concerted action *in vivo* in the genome context and at different types of repetitive elements. Data on ESCs with individual and combined Dnmt knockouts indicated preferences of Dnmts for specific repetitive elements [19]. Different mathematical models were developed to simulate the kinetics of DNA methylation [20]–[25]. However, these calculations were only theoretical or based on only scarce sequencing data. Moreover, most data sets used were based on the bisulfite analysis of only one DNA strand and/or did not discriminate between single Dnmt functions.

In this paper, we present the first comprehensive high resolution methylation analysis for both DNA strands of distinct classes of repetitive elements and four single copy genes known to be methylated in ESCs. Using hairpin linker technology combined with 454 sequencing, we generated individual patterns from embryonic fibroblasts, liver cells, wt ESCs and ESCs depleted for Dnmt1, Dnmt3a, Dnmt3b, Dnmt3a/b, Dnmt3L, Np95 and Suv39h. The Dnmt KO data sets were then used to calculate Dnmt efficiencies with improved hidden Markov models (HMM), extending previous elegant approaches by Sontag *et al.* and Genereux *et al.* [20], [25]. The comparative prediction/validation analysis documents a more differentiated view on the relative contributions of individual Dnmts for maintenance and *de novo* methylation of CpG positions. In addition, the comprehensive hairpin technology allowed us to unambiguously identify the presence and the patterns of non-CpG methylation.

## Results

### Outline and Quality Monitoring of the Hairpin-Bisulfite Sequencing Strategy

We designed specific hairpin linker protocols to amplify representative fragments of the four major classes of repetitive elements and four single copy genes from bisulfite treated mouse DNA to obtain the methylation pattern of complementary CpGs (CpG dyads) (see for a general scheme Figure S1 and for details see Materials and Methods S1). The repetitive elements selected were i) major Satellites, ii) IAPLTR1, a class of LTR-retrotransposons, iii) the 5′ untranslated region of L1Md_Tf, a long interspersed element (LINE) and iv) B1 elements, representing a class of short interspersed elements (SINEs) (see Figure S2 for locations and Table S1 for references). In this paper, we conveniently refer to the specific repetitive elements as mSat, IAP, L1 and B1, respectively. In addition, we established assays for four single copy genes: alpha feto protein (Afp), testis expressed gene 13 (Tex13), insulin growth factor 2 (Igf2) and Small nuclear ribonucleoprotein-associated protein N (Snrpn). Following amplification, PCR products were sequenced on a 454 GS-FLX sequencer with an average read length of 200–400 bp covering 3 to 12 CpG dyads of the respective amplicons.

The addition of a hairpin linker containing several unmodified cytosines allowed us to directly monitor the bisulfite conversion rates per sequenced molecule. In the linker sequences, the conversion rates ranged from 97,9 to 99,9%, with only B1 showing conversion rates below 98.7%, probably due to the more degenerate sequence composition and occasional back-folding (Table S2).

**Figure 1:**
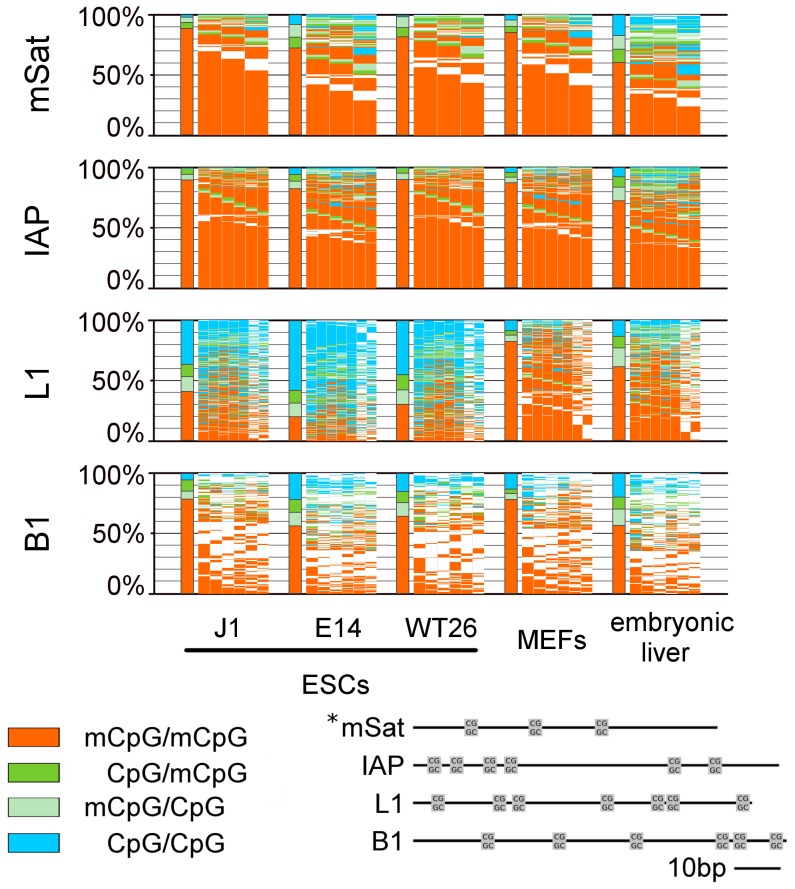
DNA methylation pattern of CpG dyads at repetitive elements in WT ESCs, MEFs, and embryonic liver. The bars sum up the DNA methylation status of all CpG dyads. The map next to the bar represents the distribution of methylated sites. Each column shows neighboured CpG dyads, and each line represents one sequence read. The reads in the map are sorted first by fully methylated sites and then by hemi-mCpG dyads. Red - fully methylated CpG dyads, light green and dark green - hemi-mCpG dyads on the upper and lower strand, blue - unmethylated CpG dyads, white - mutated or not analysable. *This picture shows the distance of the CpG dyads to each other. In MEFs and embryonic liver, hemimethylated sites are equally distributed all over the repetitive elements, whereas in ESCs elements specific differences in the amount of hemimethylated sites become obvious.

In contrast to conventional single strand bisulfite sequencing, hairpin bisulfite sequencing allows one to unambiguously distinguish between unmethylated and mutated CpG sites. We identify mutated CpGs in all repetitive elements to various extents (see white positions in Figure 1). A particular abundance of mutated CpGs was found in B1 elements with 44% of CpGs being mutated to TpG (Figure S3a). Previous single strand bisulfite sequencing accounted such TpGs as unmethylated positions estimating the total methylation of B1 elements to be only 10% [26]. When correcting for mutated sites, we find B1 elements to be methylated up to 80% in wt cells (Figure S3b).

### Analysis of the Methylation Symmetry at CpG Dyads

**Figure 2:**
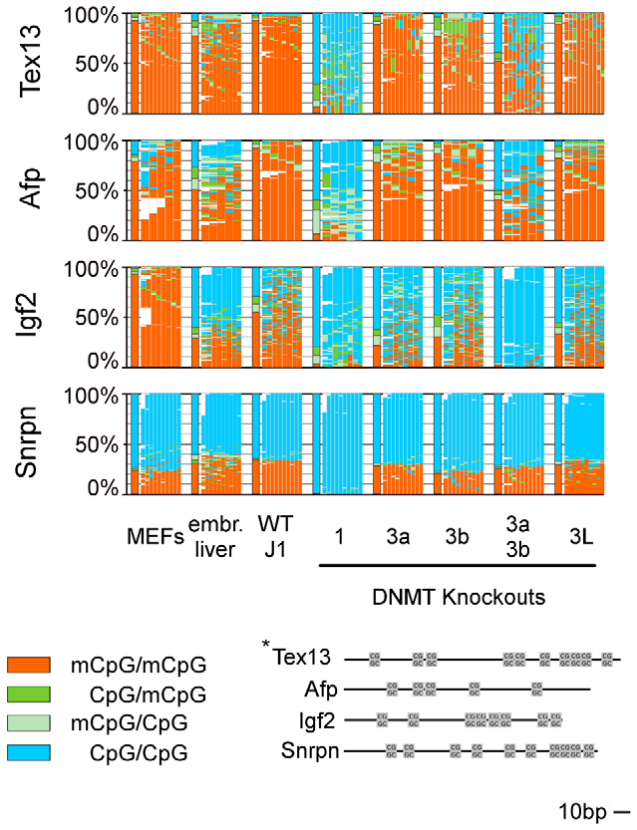
Methylation pattern of 4 single copy genes in WT ESCs, embryonic liver, and MEFs and in Dnmt KO ESCs. For detailed description see legend Figure 1. In Dnmt1 KO all analyzed regions show a hypomethylated state. In Dnmt3a/3b KO sequence specific differences become obvious.

Following a precise alignment to reference sequences using BiQAnalyzerHT [27] and the back mapping of complementary CpG positions, we first compared the DNA methylation patterns between mouse wt ESC lines, mouse embryonic liver and cultured mouse embryonic fibroblasts (MEFs) (Figure 1 and Figure 2). In general, mSat, IAPs, B1, Afp and Tex13 are highly methylated in all wt ESC and somatic cells (62–95%), whereas L1 is highly methylated in somatic cells, but only 30 to 52% in ESCs. Igf2 shows in all cell types an intermediate methylation level (Figure S3b).

The hairpin-bisulfite method allows to unambiguously discriminate between unmethylated, hemimethylated and fully methylated CpG dyads. Since it is believed that the maintenance of methylation is very stable and occurs semi-conservative, hemimethylated sites should occur very rarely. The analyses of human DNA showed that hemi-mCpGs occur between 4.8% (sperm) and 20.8% (leukocytes) at human LINE1 elements [24], [28] and 7% hemi-mCpG at human Satellite 2 sequences in different tissues [29].

We found in the analysed mouse cells a range of 1 to 25% of all CpGs in a hemimethylated status across all amplicons (Figure 1 and Figure 2). In differentiated cells, hemimethylated sites occur equally distributed across the analysed sequences - MEFs show the lowest and least variable rate of hemi-mCpG (5,8 to 12% of all methylated CpG dyads) among the analysed elements and in embryonic liver an overall high amount of hemi-mCpGs is detected (16,2 to 30,6% of all methylated CpG dyads) (Figure S3c).

Contrarily, in ESCs the degree of hemimethylation is much more variable at the different types of repetitive elements. While hemimethylation levels are low for IAPs (9,3 to 12,5%) Tex13, Afp and Snrpn (3,8 to 7%), more than 35% of methylated CpGs in L1 and 22% at Igf2 are hemimethylated. Moreover, the extent of hemi-mCpGs at mSat and B1 greatly varied between the three wt ESC lines, but the general tendencies for particular elements are maintained.

The almost exclusive fully or unmethylated patterns of the imprinted gene Snrpn (and H19, data not shown) show the stable maintenance of two non-equilibrium states. The imprinted genes are very important internal controls showing i) that the enzymes responsible for full maintenance are present and fully functional ii) that the occurance of hemimethylated states in other genes/elements is not simple due to an increase of cells analysed in S-phase (i.e. incompleted replications states) in fast dividing ESCs.

### Effects of Dnmts Loss on Overall DNA Methylation

**Figure 3:**
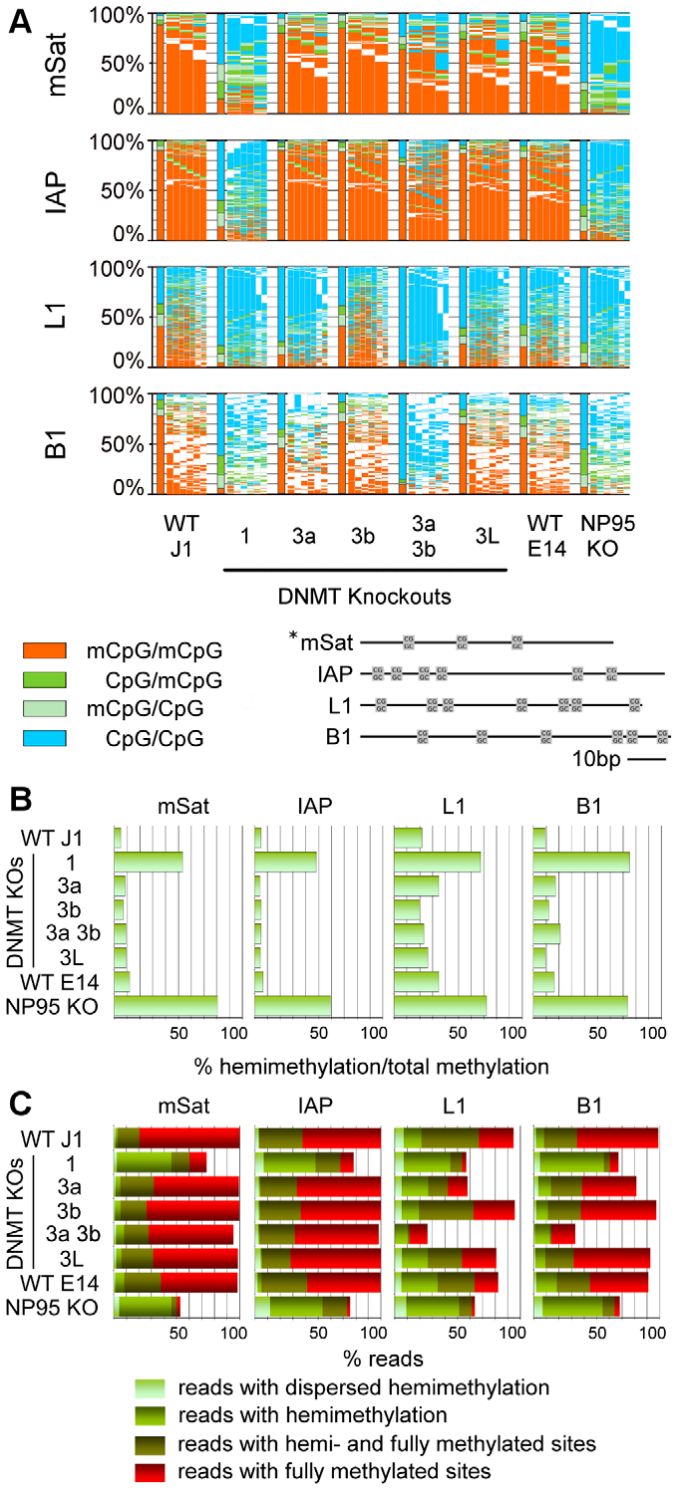
Methylation pattern of CpG dyads at repetitive elements in ESCs depleted for Dnmts or factors of the methylation machinery. A: Methylation pattern map. For detailed description see legend Figure 1. In Dnmt1 KO all elements show a hypomethylated state. In Dnmt3a/3b DKO, element specific differences are obvious. Np95 KO (corresponding WT E14) shows a hypomethylated state, comparable to the Dnmt1 KO. B: Relative amount of hemimethylated CpGs. The relative amount of hemimethylated CpGs is the ratio between hemimethylated CpGs and the overall methylation. C: Distribution of methylated/hemimethylated CpG sites along reads. The fraction of reads showing fully methylated sites (+/−unmethylated sites) is colored red. The fraction of reads with hemi- and fully methylated (+/−unmethylated sites) sites is shown in dark green. Reads with hemimethylated site (+/−unmethylated sites) are colored green. Reads in light green have hemimethylated sites on the upper and lower strand at the same time (dispersed hemimethylation).

Next, we analysed the contributions of Dnmts and cofactors for the maintenance of the methylation pattern by comparing hairpin-bisulfite sequence data of ESCs mutated for Dnmt1, Dnmt3a, Dnmt3b, Dnmt3a and 3b (DKO), Dnmt3L and Np95 (UHRF1), respectively (Figure 2 and Figure 3a).

Deletion of Dnmt1 caused a substantial reduction of DNA methylation in all analysed elements (mSat methylation was reduced by 65%, IAP by 72%, L1 by 76% and B1 by 75%, Tex13 by 82%, Afp by 75%, Igf2 by 82% and Snrpn by 99%, respectively). This tendency was also observed in previous low resolution data obtained for a subset of repetitive sequence elements [19], [30]. Our deep sequencing data however clearly shows that a small subset of sequences maintain a considerable amount of hemi- and fully methylated sites. A triple knockout cell line (TKO, data not shown) does not show any signs of DNA methylation anymore. The effects of Np95 KO at repetitive elements were very similar but not identical to the Dnmt1 KO (see also Bostick *et al.* [16]) indicating that the major activity of Dnmt1 is indeed mediated by Np95 [16], [17].

In contrast to a general hypomethylation at all elements in Dnmt1/Np95 KOs, the loss of Dnmt3 activities led to element and enzyme specific differences. While methylation at IAP, mSat, Tex13 and Afp did not greatly change in Dnmt3a or Dnmt3b single KOs, the double KO led to a clear decrease of CpG methylation at mSat (24%), IAPs (17%), Tex13 (41%) and Afp (53%). Methylation at L1 and B1 did not change in the Dnmt3b single KO, but was strongly decreased in the Dnmt3a single KO by 64% for L1 and 37% for B1. Igf2 shows decreased level for Dnmt3a and Dnmt3b single KOs (53% for Dnmt3a KO, 34% for Dnmt3b KO). For all three sequences (L1, B1 and Igf2) in the DKO, there is only minor methylation left.

Hence, while either the loss of Dnmt3a or Dnmt3b, respectively, can be compensated by the other enzyme at IAPs, mSat, Afp and Tex13 sequences, the situation is more complex at B1, L1 and Igf2. Finally, Dnmt3L also contributes to maintain a high level of methylation. In the Dnmt3L KO the loss of methylation at all regions is less extensive than in the Dnmt3a/3b DKO, arguing for a stimulatory effect of Dnmt3L on both *de novo* Dnmts. Note that Dnmt3L KO cells were at passage 15 and underwent already almost twice the amount of replications than the Dnmt3a/b DKO (passage 8).

### Loss of Dnmt1 and Np95 KO Leads to an Increase of Hemi-mCpG Sites

For all sequences, we observe a strong increase in the relative amount of hemi-mCpGs (in regard to total methylation) in Dnmt1 KO and Np95 KO (Figure 2, Figure 3b), along with a huge loss of overall methylation. This observation highlights the important role of Dnmt1 in maintaining symmetrical CpG methylation. However, it is very intriguing that in both Dnmt1 and Np95 null backgrounds, we still find a considerable amount of sequences with fully methylated CpG dyads (Figure 2, Figure 3a and 3c).

Chromosomal sequences with hemi-mCpG sites on only the upper or lower strand, respectively, were found frequently, compared to sequences with (dispersed) hemi-mCpG sites on both upper and lower strands. Such dispersed hemimethylation was found in WT ESCs in <2% of mSat, <3,4% of B1, <4,5% of IAP and <7,2% of L1 reads, respectively (see Figure 3c). Note that in Dnmt1KO and/or Np95KO ESCs dispersed hemi-mCpGs were enriched compared to WT.

In contrast to the Dnmt1 KO and Np95 KO, respectively, the abundance of hemimethylated sites does not differ between WT and Dnmt3a/Dnmt3b single KOs and double KO.

### CpA Methylation Is Pronounced at Major Satellites in ESCs

**Figure 4:**
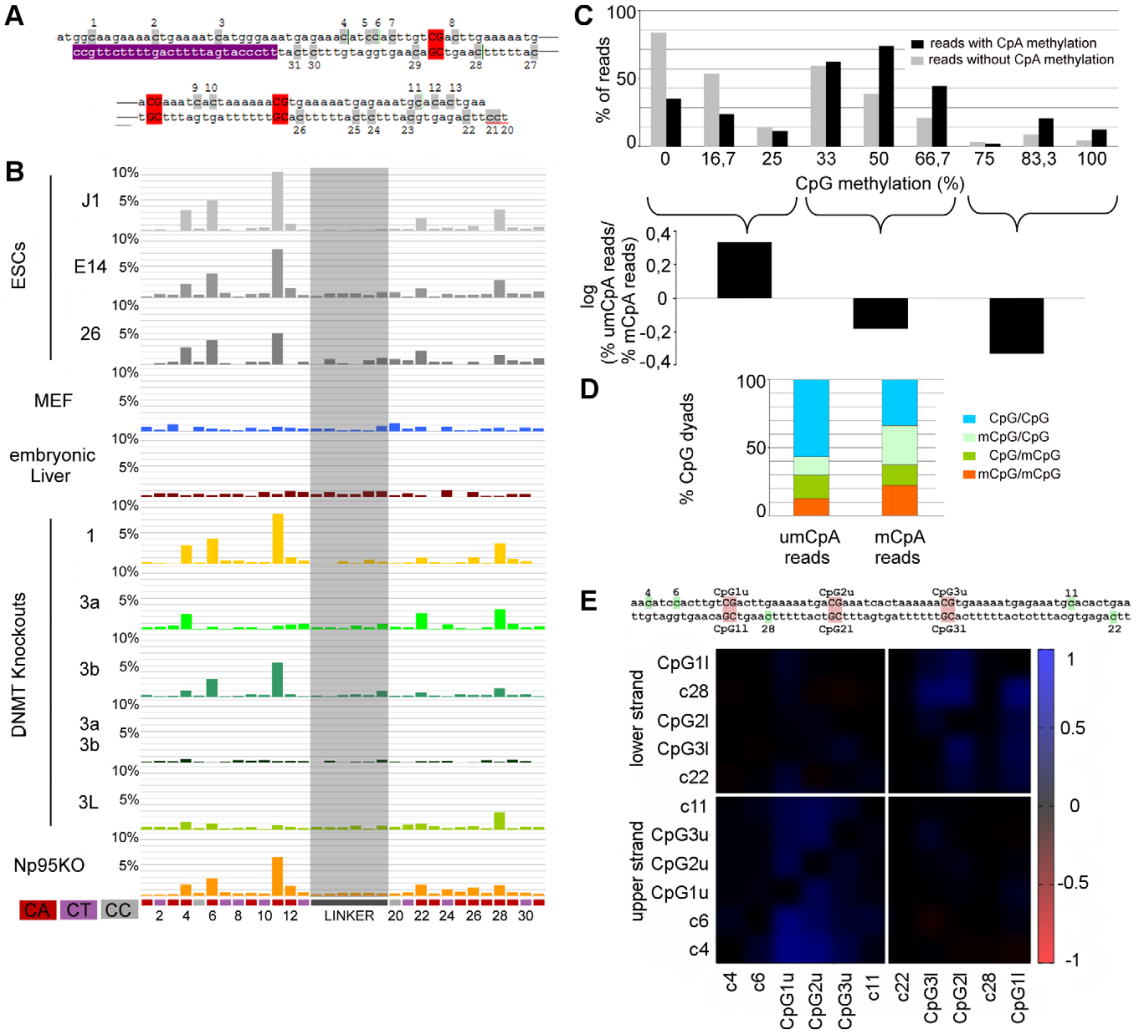
Non-CpG methylation of major satellites. A: Major satellite genomic sequence of the hairpin bisulfite PCR product. Cytosines in non-CpG context are marked grey with the corresponding number attached. CpGs are marked red. Purple shows the location of the lower primer. B: Non-CpG methylation of mESCs, differentiated cells and Dnmt and Np95 KOs at major satellites. Only CpA positions show methylation up to ten percent (position 4, 6, 11, 22 and 28). Dnmt3 family KO ESCs show decrease of CpA methylation on different sites. C: The graph represents the relative amount of reads per CpG methylation level: grey - reads showing no CpA methylation, black - reads showing CpA methylation. The reads were grouped into three fraction by CpG methylation level (0–25%, 33–66,7%, 75–100%). Reads showing CpA methylation are depleted in the fraction of reads with low CpG methylation level and enriched in reads showing 50% or more CpG methylation. D: Distribution of un-, hemi- and fully methylated CpG dyads in the reads showing CpA methylation or no CpA methylation. The fraction of reads showing CpA methylation is enriched in fully- and hemimethylated (mainly on the upper strand) CpG dyads. Interestingly, on the upper strand, we also observe the main part of CpA methylation. E: Correlation plot for cytosine methylation at Dnmt1KO ESC in mSat. Methylated CpA positions correlate to neighboured CpG positions on the same DNA strand.

**Figure 5:**
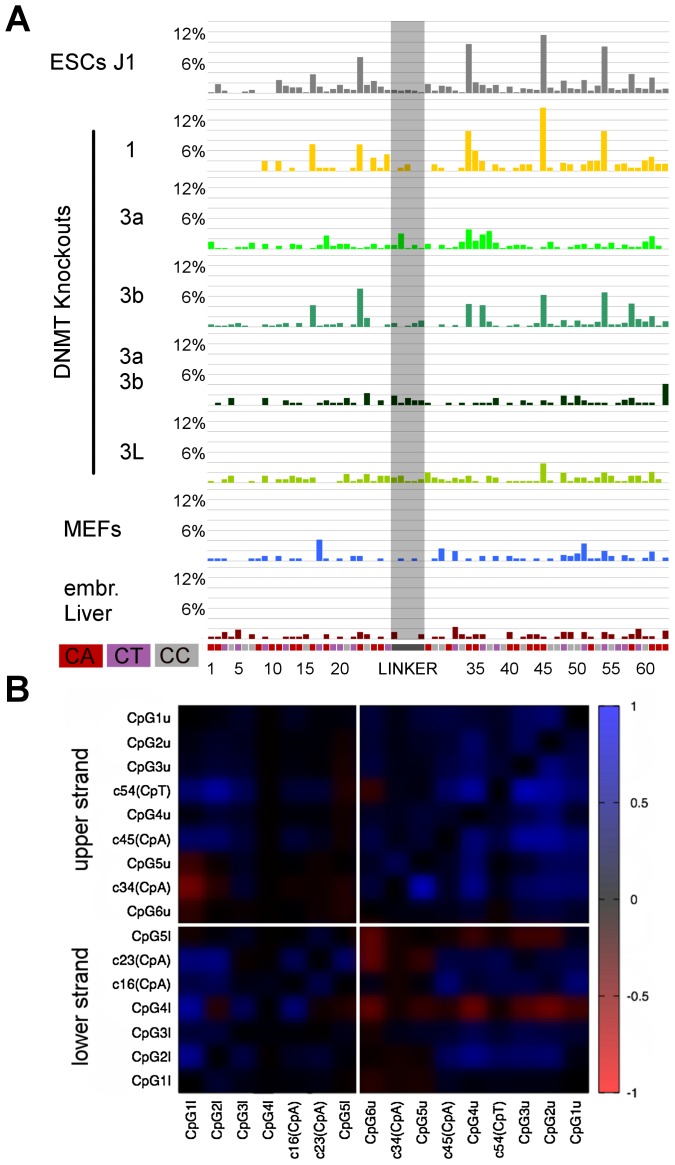
Non-CpG methylation of Afp. A: Non-CpG methylation of mESCs, Dnmt KOs and differentiated cells. Non-CpG methylation can be found at Afp at 4 CpA positions (16, 23, 34, 45) and on one CpT position (54). Dnmt3a together with Dnmt3L are responsible for this methylation, Dnmt3bKO shows only slight effect. B: Correlation plot for cytosine methylation at Dnmt1KO ESCs at Afp. Methylated CpA positions mostly correlate to methylated neighboured CpG positions.

The double stranded hairpin sequencing data allowed to unambiguously assign cytosine methylation outside of CpGs. We identified clear non-CpG (mostly CpA) methylation in mSat sequences and the Afp gene in WT ESC lines (Figure 4a and 4b, Figure 5a). This non-CpG methylation is much less pronounced, more sporadic, less position dependent or barely detectable at the other elements (Figure S4). In mSat and Afp amplicons, respectively, cytosines at five non-CpG positions showed a significant methylation (in 6–12% of all reads) clearly above the conversion background of 1.1% (as defined by linker sequence conversion, see above). Most bisulfite unconverted (methylated) positions are found in the CpA sequence context. Interestingly, in most of the sequence reads only one single CpA methylated position was detected; such that 75% for mSat and 55% for Afp of J1 reads had clear single CpA methylation (Figure S5). Finally, our data confirm that methylated cytosines (above technical background) outside of the CpG context are not detectable in differentiated cells (embryonic liver and MEFs (Figure 4b, Figure 5a).

### Dnmt3a, 3b Together with 3L Mediate Non-CpG DNA Methylation in Major Satellites

By comparing the presence of methylated cytosines in non-CpG context between wt and the different KO ESC lines (Figure 4b, Figure 5a), we found that in Dnmt1 KO the methylation at all non-CpG positions remained unchanged despite the greatly reduced CpG methylation level. Notably, we found enrichment in CpG methylation at sequences showing non-CpG methylation (Figure 4c, 4d). Moreover, by correlating CpA methylation to CpG methylation in the Dnmt1 KO, we found that CpA methylation is highly linked to neighboured methylated CpG positions at mSat and Afp (Figure 4e and Figure 5b)

In Dnmt3a/3b DKO, CpA methylation above background is completely absent and surprisingly for mSat Dnmt3a and Dnmt3b single KO showed different pattern of CpA methylation. Whereas CpA methylation at position 6 and 11 is greatly reduced in a Dnmt3a KO, the loss of Dnmt3b diminishes CpA methylation at position 4, 22 and 28. Interestingly, the loss of Dnmt3L greatly reduces the methylation at most positions. At the Afp gene, non-CpG methylation also strictly depends on Dnmt3a/3b in combination with Dnmt3L - although here Dnmt3b apparently plays a less important role.

Together these findings clearly point towards a position specific exclusive Dnmt3a and 3b mediated CpA methylation guided by Dnmt3L.

### Suv39h KO Decreases CpG Methylation Level at mSat, but Has No Influence on CpA Methylation

The Suv39h1/2 mediated modification of histone H3 at position 9 was reported to influence the targeting of DNA methylation. We therefore included ESCs and MEFs with KO for Suv39h1 and Suv39h2 (Suv39dn) in our analysis for the repetitive elements. Suv39dn ESCs were reported to lack H3K9 trimethylation and Dnmt3b localisation at pericentric heterochromatin. Lehnertz *et al.* reported reduced DNA methylation (by southern blot) at mSat in Suv39h KO but not at minor Satellites or a C-type retrovirus [31]. Our hairpin bisulfite analysis confirmed this finding on a sequencing basis. DNA methylation at major satellites is reduced by 20% in Suv39dn ESCs, but not at B1, IAP and Line1 elements. Surprisingly, the effect on mSat methylation is almost absent in dnMEFs, which retain 95% of wt methylation (Figure S6a).

Finally, despite of the proposed interaction of Suv39h with Dnmt3b at mSat, we do not observe any influence of the Suv39h absence on CpA methylation, particularly not at the Dnmt3b specific positions 4, 22 and 28 (Figure S6b).

### Hidden Markov Model Predicts Methylation Efficiencies of Dnmts

**Figure 6:**
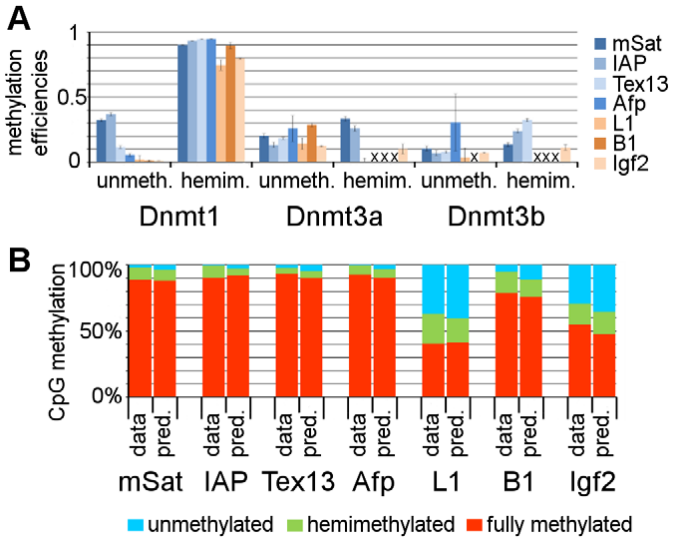
Estimation of Dnmt efficiencies using a Hidden Markov Model. A: Dnmt efficiencies. In this diagram the methylation efficiencies for all three Dnmts are given. For all three Dnmts, we discriminate between the activity to methylate unmethylated CpG positions (unmeth.) and to methylate hemimethylated CpGs (hemim.). For Dnmt1, we find element specific methyltransferase activity at unmethylated CpG positions. At L1 and Igf2 sequences, Dnmt1 shows reduced activity at hemimethylated CpG sites. For Dnmt3a/3b, we find for some elements higher activity at hemimethylated positions. For some elements, the efficiencies are not given, since standard deviations were too high (marked with a cross) (for values see Table S3). B: Prediction of WT methylation. Taking the efficiencies, estimated in the HMM with the KO data, we predicted the methylation of the WT ESC line. This prediction (pred.) fits to the real (experimentally observed) methylation data (data).

The precise determination of fully methylated, hemimethylated and unmethylated CpG dyads in the comparative data set of some 28.000 sequences (around 146.000 CpG dyads) including wt ESCs and Dnmt KOs allowed us to calculate the element specific methylation efficiencies for the different catalytically active Dnmts in a modified version of the linear HMM proposed by Sontag *et al.* [20]. We computed maximum likelihood estimates for both methylation efficiencies on unmethylated and hemimethylated CpG dyads separately. As opposed to previous calculations [24], [25], we used the information of Dnmt KOs, to combine these in a single model to obtain Dnmt specific efficiencies at unmethylated and hemimethylated CpG dyads. Furthermore, we did not assume that steady-states are reached in the KO ESC lines. Instead, we estimated the amount of cell generations and inferred parameters during the transient phase of the system, since at least Dnmt3a/3b DKO shows a progressive loss of DNA methylation with increasing passage number [32]. The estimated efficiencies with standard deviations are given in Figure 6a and Table S3. The approximated standard deviations showed that for Dnmt1 efficiencies were accurately estimated for all sequences. For Dnmt3a and Dnmt3b standard deviations are too high for a conclusion at L1, B1 and Afp.

To substantiate the appropriateness of our model and the accuracy of our estimated methylation efficiencies, we predicted the DNA methylation level for the parental wt ESC line (Figure 6b and Table S4). Indeed, we found good predictions for all elements, with maximum error rates of 1.7% (mSat), 4,1% (IAP), 3.9% (Tex13), 2.7% (Afp), 4,1% (L1), 5,9% (B1) and 7,1% (Igf2).

Based on the HMM calculations, we find a high activity of Dnmt1 on hemimethylated CpG dyads. This is 90% or higher for mSat, IAP, Tex13, Afp and B1, but remarkably lower for L1 and Igf2. Furthermore, we found clear evidence for *de novo* methylation activity of Dnmt1 *in vivo*. However, it differs for the classes of repetitive elements and single copy genes. While Dnmt1 does not show a remarkable *de novo* methylation activity (<0.02) for L1, B1 and Igf2, this activity is apparent at IAP, mSat, Tex13 and Afp with calculated efficiencies of 0.36, 0.32, 0.12 and 0.06, respectively (Figure 6a, Table S3). Interestingly, for the *de novo* methyltransferases Dnmt3a and Dnmt3b, we observe a higher efficiency at hemimethylated sites for some targets, which contrasts *in vitro* derived data [8], [9].

## Discussion

In our work, we present the first high resolution DNA bisulfite methylation analysis of different repetitive elements and selected single copy genes in double stranded DNA. Comparative analysis of wt and KO mouse ES lines revealed detailed insights in the relative contributions of Dnmts to CpG dyad and CpA methylation. In a HMM, we predicted the relative contribution of Dnmts to methylate unmethylated and hemimethylated CpG dyads.

### Efficiencies of Dnmts

According to the changed methylation pattern observed in our hairpin-bisulfite analysis and the methylation probabilities of Dnmts estimated with our HMM, the analysed elements can be grouped into three different classes: (i) The imprinted genes (Snrpn and H19 (data not shown)), which exclusively depend on Dnmt1 for maintenance methylation. (ii) IAP, mSat, Tex13 and Afp, which are mainly dependent on Dnmt1 (methylation activity at unmethylated and hemimethylated CpG positions) and show minor effect in the Dnmt3a/3b DKO and (iii) L1, B1 and Igf2, which need Dnmt1 (only methylation activity at hemimethylated CpG positions) and Dnmt3a/3b to work cooperatively. Thus, the HMM model shows that the maintenance contribution for specific genomic regions is clearly distinct from the Dnmt(s) contributions for the *de novo* acquisition of methylation. Here, the methylation of IAP and L1 is reported to be dependent on either Dnmt3a or Dnmt3b, whereas mSat need Dnmt3b and B1 elements Dnmt3a [33].

Our HMM applied to estimate methylation efficiencies significantly extends previous modelling approaches and allows to draw functional conclusions. First, by separating the effects of all three Dnmts based on KO data, we could estimate probabilities to methylate unmethylated or hemimethylated CpG positions for each enzyme independently. Second, our experimental strategy allowed to precisely assign CpG dyads and to account for experimental measurement errors (bisulfite conversion and mutation errors) as well as the number of cell divisions (passages). Third, we employed numerical techniques to infer optimal methylation efficiencies since analytic solutions of our more complex model are infeasible. By integrating all these parameters, we could functionally extend the previous models developed by Genereux *et al.* and Sontag *et al.* [20], [25]. Moreover, beyond prediction, our validations (see Figure 6, Table S4) demonstrate the appropriateness of the model at least for mSat, IAP, Tex13 and Igf2. For B1, L1 and Afp the prediction is very accurate even though the efficiencies of Dnmt3a/3b are difficult to estimate. In contrast to our estimations based on biological Dnmt KO data, a recently published model discriminates between the Dnmts only using theoretical considerations for the Dnmts on WT methylation pattern [34]. However, in this model the authors estimate the processivity of the Dnmts. It will be interesting to adapt their model, using our biological Dnmt KO data.

For Dnmt1, our HMM indicates a significant methylation probability at unmethylated CpG dyads (*de novo* methylation) in ESCs (up to 22%), depending on the repetitive element/sequence. *In vitro* experiments analysing the methylation activity of Dnmt1 show 2 to 50 fold higher preference for hemim-CpG dyads, dependent on the substrate or conditions [35]. *In vivo*, we find 2.5 to 90 fold higher preference for hemimethylated CpGs. Interestingly, pre-existing methylated sites *in vitro* were shown to enhance the *de novo* methylation efficiency of Dnmt1 [12],[36]–[38]. Our data corroborate these observations *in vivo*, linking an increased methylation to a higher *de novo* methylation activity of Dnmt1 (CpG methylation and methylation activity at unmethylated CpGs is higher at IAP, mSat, Tex13, Afp than both at L1, B1 and Igf2). Differential regulation of the CXXC domain binding capacities at the different sequences could influence the *de novo* methylation activity of Dnmt1 [39]. The fidelity for Dnmt1 to methylate hemi-mCpG dyads was shown to be 95% to 96% *in vitro* [12]. Our HMM predicts fidelities of methylating hemi-mCpGs for IAP, mSat, B1, Tex13 and Afp of 90 to 95%, which fits quite well with the *in vitro* data. However, at L1 and Igf2, the fidelity decreases to less than 80%. This lower fidelity at hemim-CpGs might arise from the presence of 5hmC at L1 elements and Igf2 (see discussion next chapter, reference [40], Figures S7 and S8). 5hmC could hereby not only influence maintenance methylation but also *de novo* methylation activity, which is enhanced by 5mC content but presumably not 5hmC.

For Dnmt3a and Dnmt3b, we found significant “maintenance” methylation activity. However, the ratio of *de novo* and maintenance methylation contributions differs across sequence elements. Such context dependent effects were not addressed in former *in situ* and *in vitro* modelling studies and may become only evident in the native chromatin context. Since *in vitro* Dnmt3a and 3b appear to methylate independent of the methylation status of the CpG dyad, the high contribution of Dnmt3a and 3b to maintain full methylation at CpG dyads following replication might be attributed to targeted and enhanced *de novo* activity stimulated by the presence of CpG methylation density. Some studies show that Dnmt3a/3b can strongly bind to nucleosomes containing methylated DNA [41], [42]. By this Dnmt3a/3b could be triggered to “*de novo*” methylate hemimethylated sites following replication to maintain full methylation in the absence of Dnmt1.

### Distribution and Specificity of CpG Hemimethylation and the Relation to 5hmC

Our experimental approach allowed us to unambiguously assign DNA methylation in total at about 280.000 CpG dyads at 4 repetitive elements and 4 single copy genes on both DNA strands. Besides a general prevalence for symmetrical methylation, we found a substantial portion of hemimethylated CpG dyads in all cell types analysed. The presence of such hemimethylated CpGs can be explained by three different mechanisms: i) the improper recognition of modified cytosines and/or impaired maintenance activity, ii) the selective *de novo* methylation or iii) active DNA demethylation.

In MEFs and embryonic liver, we find different global tendencies for the occurrence of hemimethylated sites at all analysed elements. However, all three Dnmts are expressed in both cell types (Figure S9). This suggests that in embryonic liver the maintenance fidelity of Dnmts is less pronounced or alternatively DNA demethylation is more pronounced. In ESCs, we observe only at L1 sequences and Igf2 a high level of hemimethylated sites. Apparently, in ESCs, the maintenance methylation machinery is less accurate only at specific sequences and from our data we see that a strong cooperativity of Dnmt1, Dnmt3a and Dnmt3b is needed to maintain methylation at these sequences.

The analyzed cell types show strong cell cycle differences. These might be regarded as reasons for methylation differences. Cultured and fast growing ESCs, for example have been shown to be almost 5 times more likely to be in S-Phase as compared to MEFs [43], [44]. However, in our analysis ESCs show some sequences, which have the same amount of hemimethylated sites as MEFs. Furthermore, in fast growing embryonic liver we observe strong increases of reads showing hemimethylated sites next to fully methylated sites (Figure S10, fraction in dark green). We therefore regard it as unlikely that incomplete methylation can be reduced to the varying number of incomplete S-Phases in the different cell types.

5-hydroxymethylcytosine (5hmC) might contribute to the increase of hemimethylated sites either by impairing maintenance methylation or inducing active DNA demethylation [45]–[48]. 5hmC was reported to be abundant in ESCs, but less so in cultured cells [49]–[51]. Indeed, there is a tendency that 5hmC enrichment is linked to the presence of hemimethylated CpG positions. While Igf2 and L1 regions have an increased level of hemimethylated CpGs and are enriched for 5hmC in ESCs, no ESC specific 5hmC enrichment is found at the Tex13, Afp nor IAP loci, which only show few hemimethylated sites (Figures S7 and S8).

Our HMM calculations indicate that in ESCs sequences enriched for 5hmC do not exhibit *de novo* methylation activity of Dnmt1 in contrast to 5hmC depleted sequences. Hence, 5hmC might not only impair maintenance methylation but also *de novo* methylation activity by Dnmt1. However, whether 5hmC indeed blocks Dnmt1 mediated methylation remains to be resolved. Unfortunately, 5hmC profiles cannot be distinguished from 5mC by our bisulfite based sequencing [46], [52], [53] such that the influence of 5hmC on 5mC methylation cannot clearly be assigned. Moreover, the role of 5hmC may not be of importance in some cases, since Np95 apparently recognizes and binds to 5hmC containing DNA and may moderate an effect of 5hmC on Dnmt1 recognition [54]. Finally, a selective conversion of 5mC into 5hmC at individual CpGs could cause mosaic hemimethylated situations by inducing local (hemi-) demethylation. Two general mechanisms, a direct demethylation by further oxidation to carboxylcytosine and subsequent decarboxylation as well as DNA repair coupled processes have been discussed in this respect [46]–[48]. Indeed, the intriguing presence of 5hmC at Line1 and Igf2 regions analyzed might explain the extreme mosaic pictures at these elements. If this is true the estimated *de novo* methylation rates for both elements may be completely underestimated.

To test if hemimethylated CpG positions are coupled to 5hmC, we analysed the methylation pattern of repetitive elements in the Tet1 KO ESCs. However, the Tet1 KO cells only show a 30% reduction of genome wide 5hmC [55]. We indeed see changed methylation patterns in L1 elements with a slightly increased amount of fully methylated CpG dyads (Figure S11). A combined analysis of Tet KO's (i.e. Tet1+Tet2) might further substantiate the possible link between 5hmC and the increased occurrence of hemimethylated CpG dyads.

### Control of CpA Methylation

DNA methylation outside of the CpG context was initially detected in mESCs using nearest neighbour analysis. This analysis revealed a strong prevalence for the CpA context and pointed towards a Dnmt3a dependency [56]. Recent genome wide single stranded bisulfite sequencing using Illumina short reads identified non-CpG methylation at various sequence contexts in human ESCs at rather high rates of 13–25% of all methylated Cs, mainly in CpA context [57]–[59]. In our data set covering a total of 280.000 individual CpG positions and up to 10^8^ bases, we detect non-CpG methylation at specific positions mainly in a CpA context especially confined to mSat sequences and Afp. It is possible that the primer based amplification of our analysis caused some selection against non-CpG methylation and we therefore underestimate the amount of non-CpG methylation. Still, the position dependent non-CpG methylation remains outstanding. We found that non-CpG methylation is exclusively dependent on Dnmts 3a and 3b, in concordance with recent observation in human [59]. However, we can show that both methylate non-CpG positions only in combination with Dnmt3L. Neither the absence of Dnmt1 nor Np95 altered the non-CpG methylation. Moreover, the unchanged non-CpG methylation in Suv39hdn cells reveals that the proposed protective function of H3K9 trimethylation for non-CpG methylation may not be true for mSat [60]. The sequence analysis of Dnmt1 KOs unambiguously shows that non-CpG methylation is linked to Dnmt3a and Dnmt3b mediated CpG methylation. Along this line non-CpG positions are highly co-methylated with some neighbouring CpG positions (Figure 4e, Figure 5b). A recent publication discusses a widespread unspecific non-conserved non-CpG pattern in human pluripotent cells [59]. This contrasts our findings in mouse ESCs, which suggests that non-CpG methylation is mostly locally confined to specific regions such as mSat and Afp and specific non-CpG positions. It will be important to substantiate non-symmetric methylation distribution in human by deep sequencing. Genome wide sequencing approaches at relative low coverage may easily overlook specific patterns as observed in our analysis.

Our data lets us speculate that CpA methylation results from a position specific “side reaction” of Dnmt3a and Dnmt3b stimulated by Dnmt3L. In line with this, Holz-Schietinger *et al.* show that Dnmt3L increases the processivity of Dnmt3a [13]. Finally, Dnmt3L is much more expressed in ESCs compared to somatic cells, where we do not find any evidence for CpA methylation [61].

### Conclusion

Comprehensive hairpin-bisulfite sequencing in Dnmt KO ESCs reveals a complex scenario of sequence, element and cell specific control of DNA methylation pattern at CpG dyads. Based on the sequencing data, we construct a greatly improved HMM, which reveals enzyme, cell type and genome position dependent *de novo* and maintenance methylation functions for all three Dnmts. This strongly supports previous conclusions by Fatemi *et al* 2002 and others, that *in vivo* neither *de novo* methylation can be exclusively assigned to Dnmt3a/3b nor maintenance methylation exclusively to Dnmt1 [62]. Position dependent non-CpG methylation, mainly in CpA context, occurs at major satellites and the Afp gene exclusively in ESCs. This non-CpG methylation is mediated by Dnmt3a and 3b, depends on the presence of Dnmt3L and is strongly correlated to the methylation of flanking CpG positions.

## Materials and Methods

### Hairpin-Bisulfite Sequencing

The complete protocol is provided in the SI (Materials and Methods S1). Briefly, genomic DNA was digested with an element specific restriction enzyme and the upper strand and lower strand were linked with a hairpinoligonucleotide. After bisulfite treatment an element specific PCR was performed and the resulting product was sequenced with the 454 sequencing technique.

### Estimation of Dnmt Efficiencies

We used CpG dyad methylation data on WT and DnmtKO ESCs in a hidden Markov model to estimate the Dnmt methylation efficiencies by the maximum likelihood method. A detailed description of the model is provided in the SI (Materials and Methods S1).

## Supporting Information

Figure S1 Scheme of methylation analysis with hairpin-bisulfite sequencing. Genomic DNA is digested with a restriction enzyme cutting in the region to be analyzed and a linker is ligated to the restricted site. Subsequently a bisulfite treatment follows. With primers one binding to the upper one to the lower strand the region to be analyzed is amplified and sequenced by 454 sequencing.
(TIF)

Figure S2 Location of analyzed regions. In green the location of the analyzed regions is given in relation to the whole repetitive element (A) or the single copy gene (B).
(TIF)

Figure S3 A: Fraction of mutated CpG sites in B1 elements. Averaged percentage of presumed unmethylated CpGs, which do not match after bisulfite treatment to CpG (TpG) on the complementary strand and therefore, they can be regarded as mutated (restr. = restriction site for hairpin-bisulfite analysis, mut. = heavily mutated, therefore not in analysis). B+C: DNA Methylation of WT cells at mSat, IAP, L1, B1, Tex13, Afp, Igf2 and Snrpn. B: Overall methylation level of WT cells. Overall methylation level was calculated from all CpG dyads, hemimethylated sites assessed as 0.5 methylated sites and fully methylated sites as 1 methylated site. C: Amount of hemimethylated sites splitted by occurrence of the methylation on the upper or lower strand (in percent of all methylated CpG dyads).
(TIF)

Figure S4 Methylation level of non-CpG positions at Tex13, Igf2, Snrpn, L1, B1 and IAP in ESCs, differentiated cells, different DnmtKO ESCs and Np95 KO ESCs. In grey the positions of the linker are marked. Left and right are the methylation levels of non-CpG positions on both DNA strands. For B1 also methylation at CpG positions in the reference is given, but without taken CpGs in the read into account, since at these positions we often detected CpG to CpA mutations.
(TIF)

Figure S5 CpG and CpA methylation pattern of mSat in J1 and Dnmt1KO ESCs. The map represents the distribution of methylated sites at CpA and CpG positions. Each column shows neighboured Cs (grey/black for CpAs and red/blue for CpGs), and each line represents one sequence read. Shown are only reads with CpA methylation.
(TIF)

Figure S6 Influence of Suv39h double Knockout on methylation pattern. A: Methylation pattern map of CpG dyads. The bars sum up the DNA methylation status of all CpG dyads. The map next to the bar represents the distribution of methylated sites. Each column shows neighboured CpG dyads, and each line represents one sequence read. The reads in the map are sorted first by fully methylated sites and then by hemi-mCpG dyads. Red - fully methylated CpG dyads, light green and dark green - hemi-mCpG dyads on the upper and lower strand, blue - unmethylated CpG dyads, white - mutated or not analysable. We only observe influence of the Suv39h DKO on the DNA methylation of major Satellites. B: Non-CpG methylation. We could not detect changed non-CpG methylation pattern in the Suv39h DKO cells lines.
(TIF)

Figure S7 Relative enrichment of 5hmC and 5mC in the four analyzed repetitive elements. The raw data was taken from Ficz *et al.* [*40*]. Intriguing, L1 elements are highly enriched for 5hmC and only rarely for 5mC. IAPs are only enriched for 5mC.
(TIF)

Figure S8 Enrichment profile of 5mDIP and 5hmDIP of Afp (A), Igf2 (B), Snrpn (C) and Tex13 (D). The raw data was taken from Ficz *et al.* [*40*]. Red shows the enrichment of 5hmC and blue the enrichment of 5mC. In green the position of the hairpin-bisulfite product is given. Only Igf2 shows enrichment of 5hmC in the analysed region.
(TIF)

Figure S9 RT PCR for embryonic Liver and MEFs. The analysis shows that all three Dnmt transcripts are abundant in embryonic liver and cultivated MEFs.
(TIF)

Figure S10 Occurrence of hemimethylated CpG positions in MEFs and embryonic liver. Given is the relative abundance of: reads showing either fully methylated positions (including or without unmethylated positions ( = +/−unmethylated positions)) in red, reads showing fully and hemimethylated positions (+/−unmethylated positions) in dark green, reads showing hemimethylated position (+/−unmethylated position) in green, reads showing dispersed methylation (hemimethylated sites on both strand) in light green and reads showing only unmethylated sites in blue. In embryonic liver, mainly reads showing fully and hemimethylated sites increase compared to MEFs.
(TIF)

Figure S11 CpG methylation pattern map of repetitive elements in Tet1 KO ESCs. A+B: Only in L1 minor increase in methylation can be observed in the methylation pattern map (A, description see Figure S6a)) and the overall methylation level (B). Nanog and Oct4 promotor regions are unmethylated in WT and Tet1 KO ESCs. C: The increase in methylation goes along with increased amount of fully methylated sites. The amount of hemimethylated CpG dyads stays the same and relative to total methylation decreases slightly.
(TIF)

Materials and Methods S1 Information on the analyzed Cell-lines and detailed description of the hairpin-bisulfite analysis and the hidden Markov model.
(PDF)

Table S1 Used reference sequences for the hairpin-bisulfite analysis of the repetitive elements.
(DOCX)

Table S2 Number of reads, analysed CpG positions and conversion rates of the linker sequences (the conversion rate in each sample was calculated from the linker sequence, which contains 5 to 7 unmethylated Cs) A For analyzed repetitive elements and B For analyzed single copy genes.
(DOCX)

Table S3 *De novo* and maintenance methylation efficiencies of Dnmts. Fitted efficiency values with standard deviations using maximum likelihood method, assuming that *de novo* methylation has the same probability to methylate unmethylated or hemimethylated positions. The values give the probabilities of Dnmt1, Dnmt3a and Dnmt3b mediated *de novo* or maintenance methylation per replication.
(DOCX)

Table S4 Prediction of WT methylation. Shown are the predictions for the methylation levels of the repetitive elements in WT J1 ESCs using all fitted parameters (predicted). As reference the experimental derived methylation level of WT J1 ESCs is listed (data).
(DOCX)
