# Supplementary figures and images for: In Vivo Control of CpG and Non-CpG DNA Methylation by DNA Methyltransferases
Source: PLoS Genet. 2012 Jun 28;8(6):e1002750. doi: 10.1371/journal.pgen.1002750 (PMC3386304; doi:10.1371/journal.pgen.1002750)

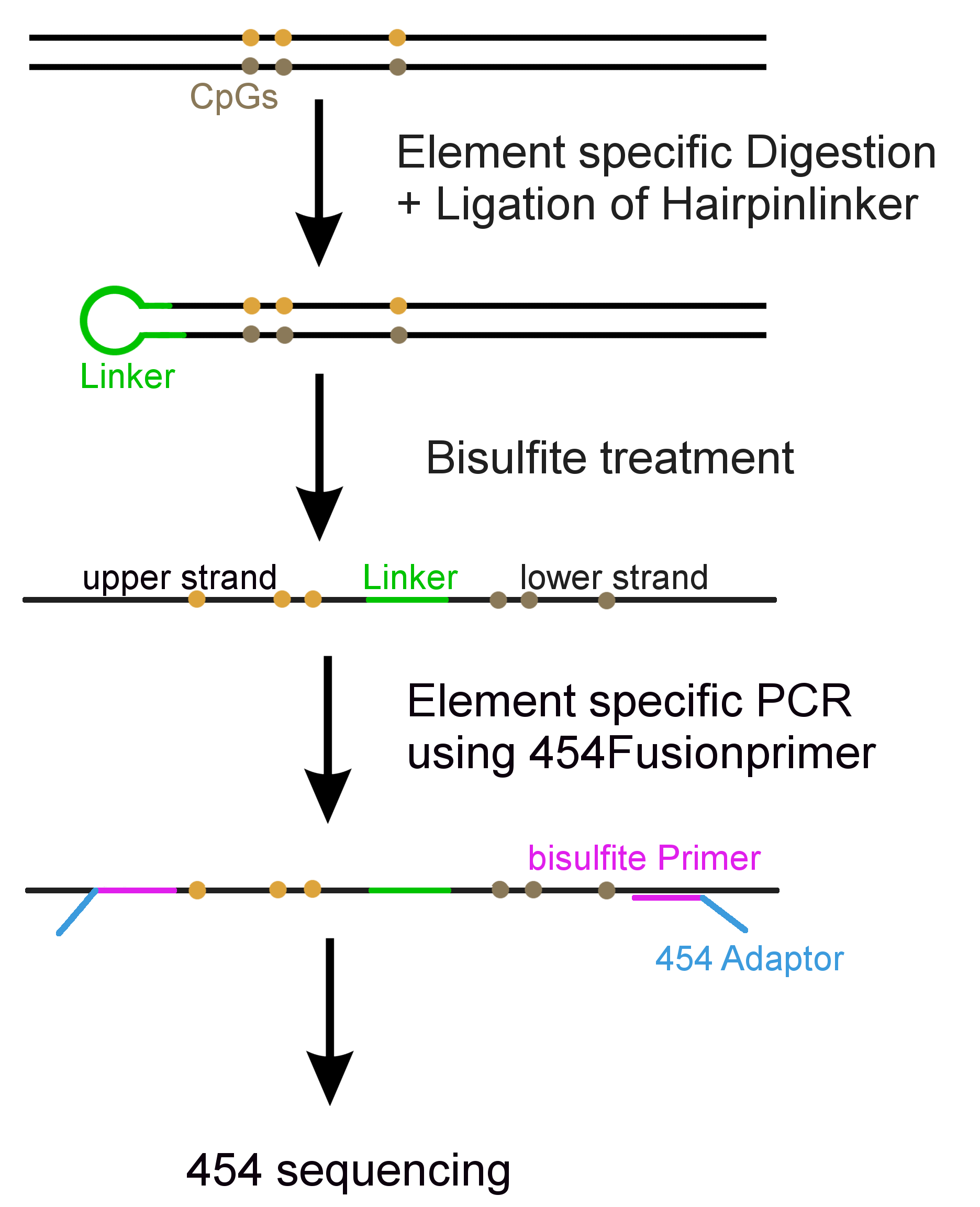

Supplement: Figure S1 — Scheme of methylation analysis with hairpin-bisulfite sequencing. Genomic DNA is digested with a restriction enzyme cutting in the region to be analyzed and a linker is ligated to the restricted site. Subsequently a bisulfite treatment follows. With primers one binding to the upper one to the lower strand the region to be analyzed is amplified and sequenced by 454 sequencing. (TIF) [file pgen.1002750.s001.tif]

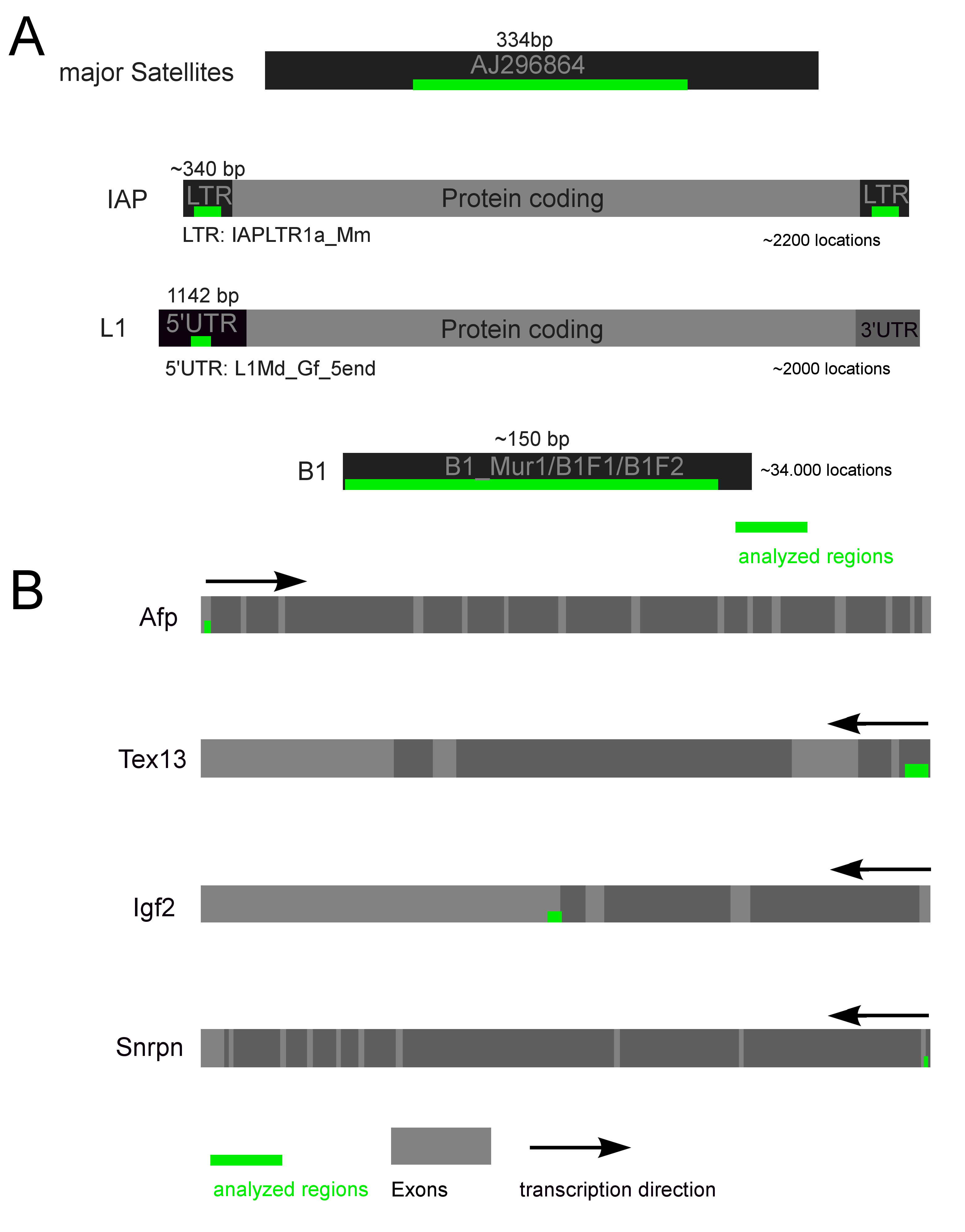

Supplement: Figure S2 — Location of analyzed regions. In green the location of the analyzed regions is given in relation to the whole repetitive element (A) or the single copy gene (B). (TIF) [file pgen.1002750.s002.tif]

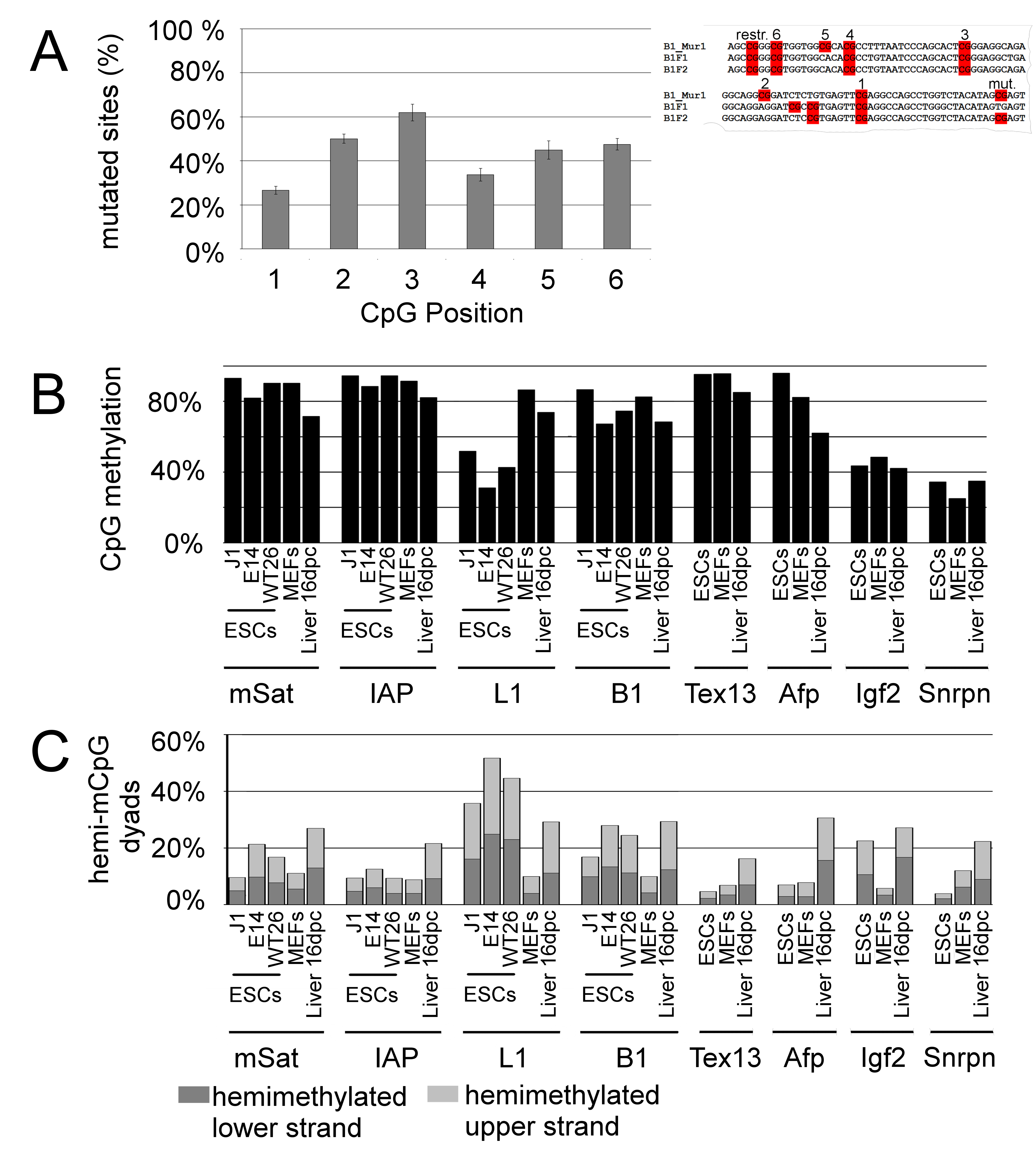

Supplement: Figure S3 — A: Fraction of mutated CpG sites in B1 elements. Averaged percentage of presumed unmethylated CpGs, which do not match after bisulfite treatment to CpG (TpG) on the complementary strand and therefore, they can be regarded as mutated (restr. = restriction site for hairpin-bisulfite analysis, mut. = heavily mutated, therefore not in analysis). B+C: DNA Methylation of WT cells at mSat, IAP, L1, B1, Tex13, Afp, Igf2 and Snrpn. B: Overall methylation level of WT cells. Overall methylation level was calculated from all CpG dyads, hemimethylated sites assessed as 0.5 methylated sites and fully methylated sites as 1 methylated site. C: Amount of hemimethylated sites splitted by occurrence of the methylation on the upper or lower strand (in percent of all methylated CpG dyads). (TIF) [file pgen.1002750.s003.tif]

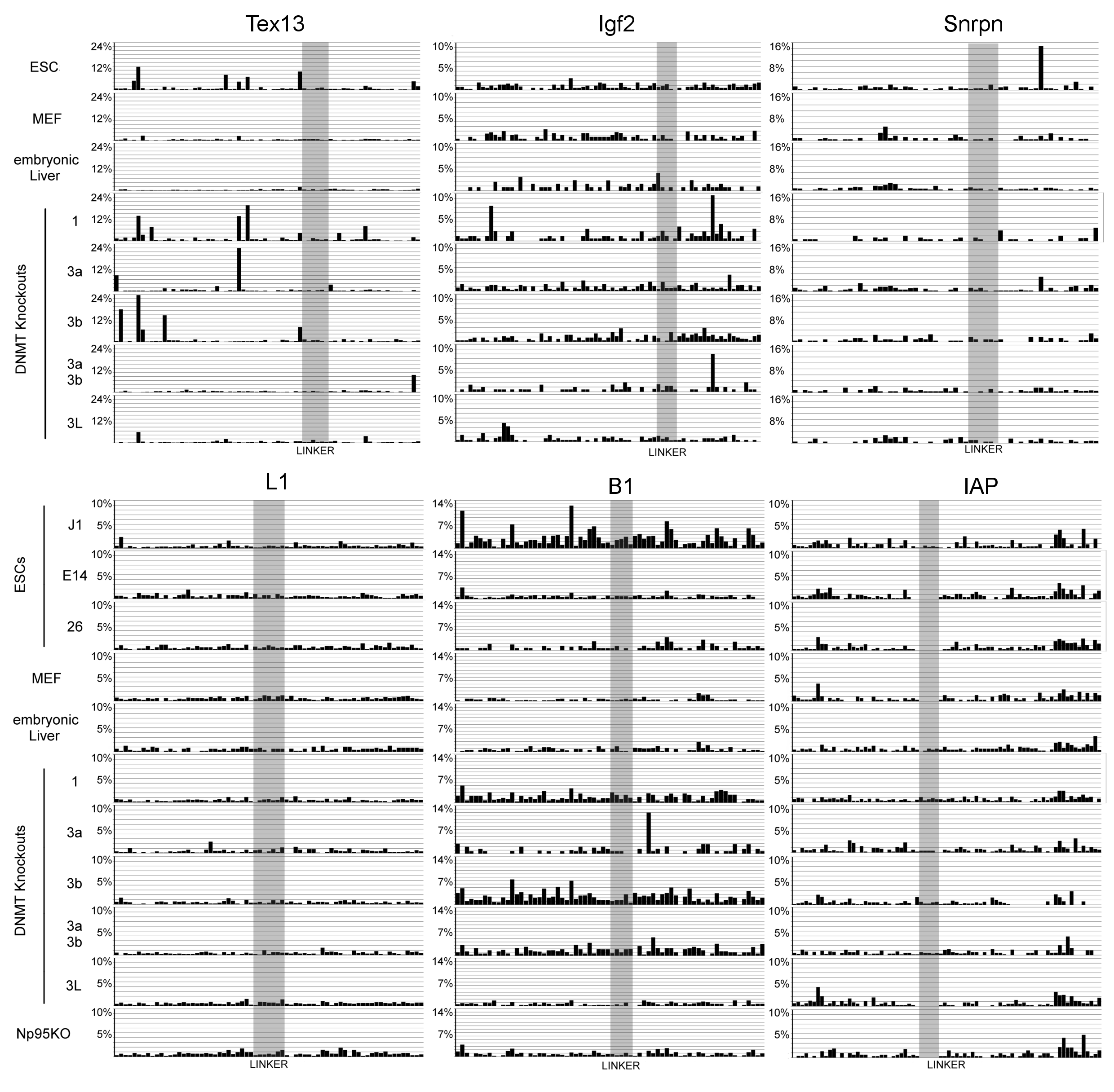

Supplement: Figure S4 — Methylation level of non-CpG positions at Tex13, Igf2, Snrpn, L1, B1 and IAP in ESCs, differentiated cells, different DnmtKO ESCs and Np95 KO ESCs. In grey the positions of the linker are marked. Left and right are the methylation levels of non-CpG positions on both DNA strands. For B1 also methylation at CpG positions in the reference is given, but without taken CpGs in the read into account, since at these positions we often detected CpG to CpA mutations. (TIF) [file pgen.1002750.s004.tif]

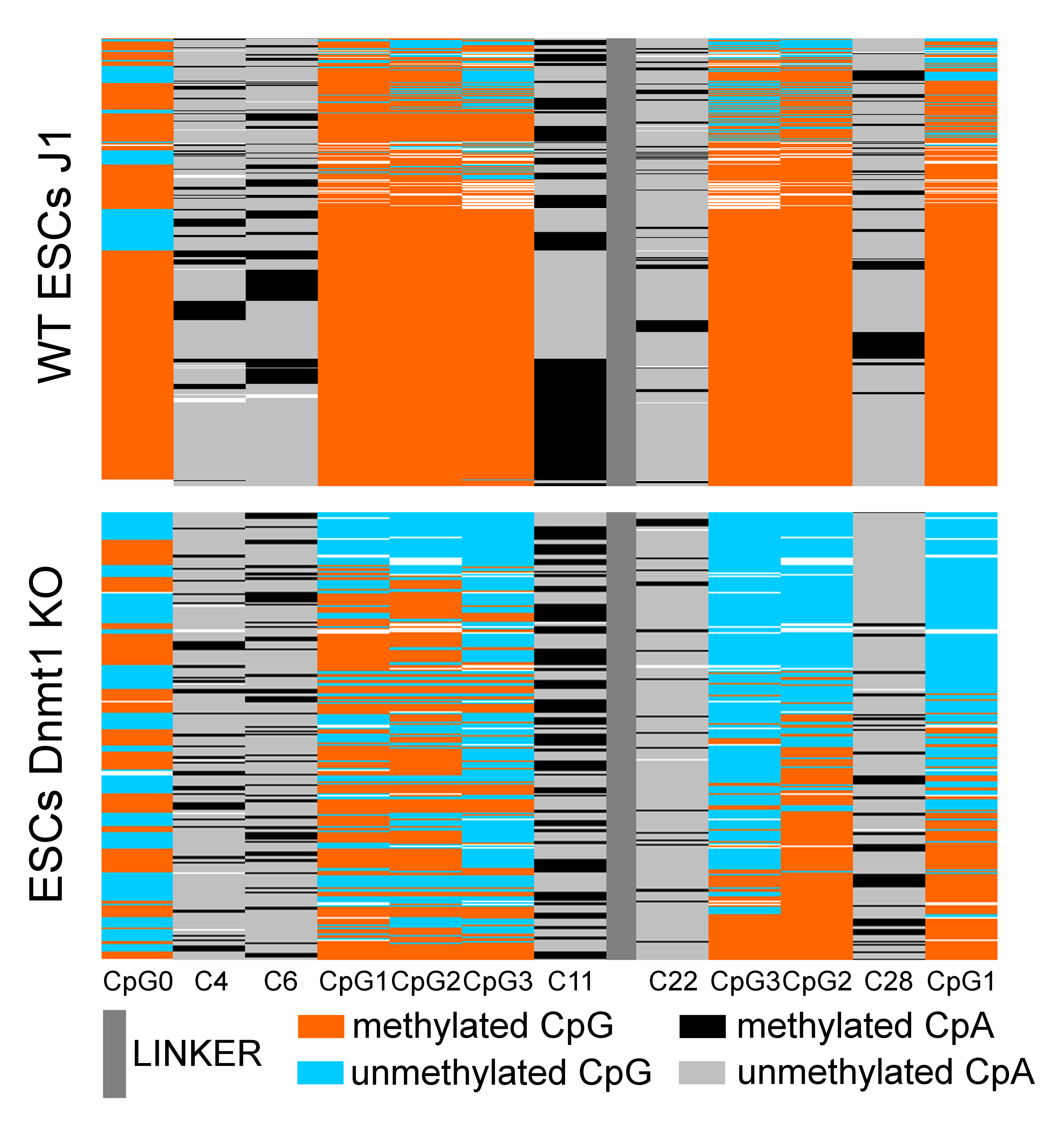

Supplement: Figure S5 — CpG and CpA methylation pattern of mSat in J1 and Dnmt1KO ESCs. The map represents the distribution of methylated sites at CpA and CpG positions. Each column shows neighboured Cs (grey/black for CpAs and red/blue for CpGs), and each line represents one sequence read. Shown are only reads with CpA methylation. (TIF) [file pgen.1002750.s005.tif]

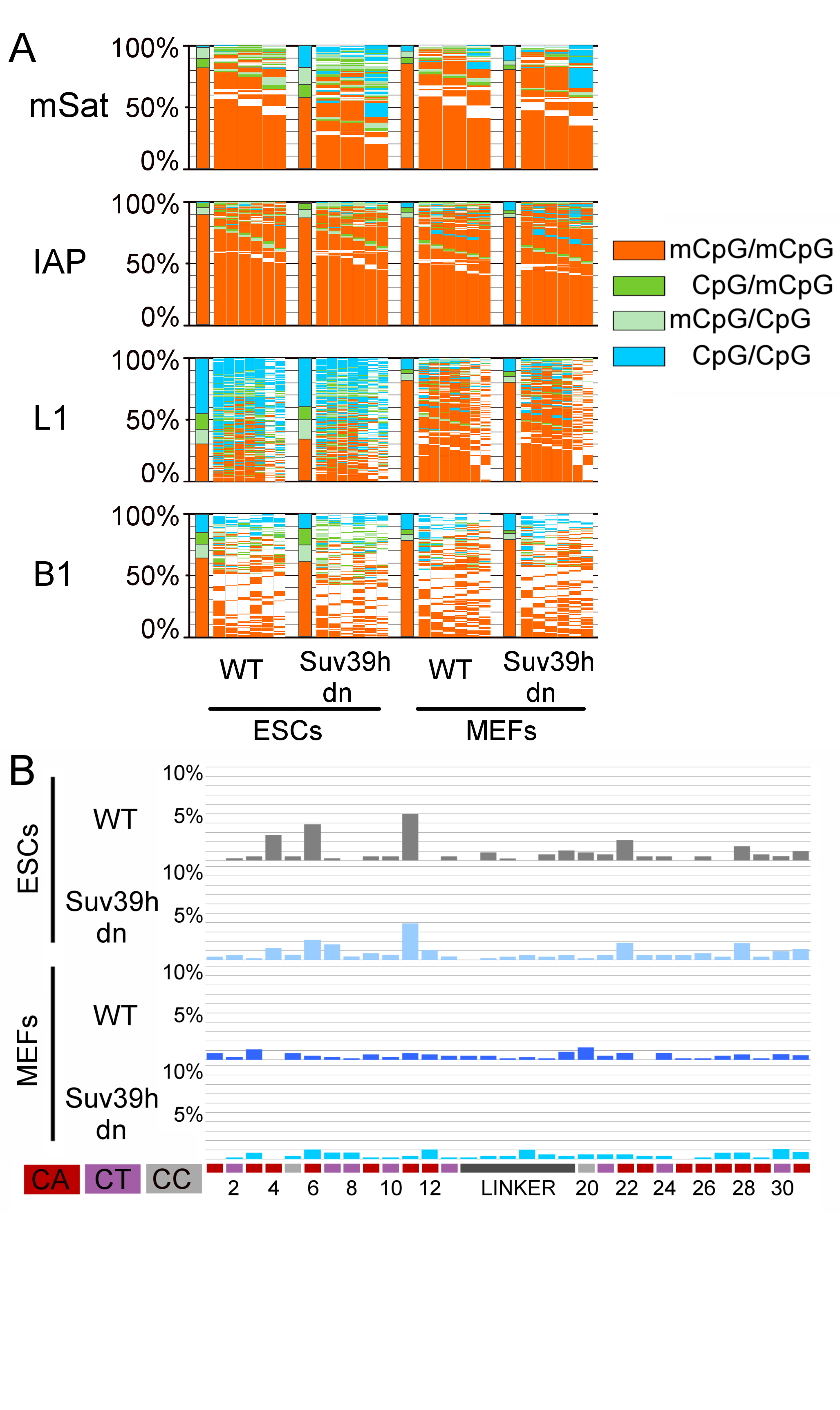

Supplement: Figure S6 — Influence of Suv39h double Knockout on methylation pattern. A: Methylation pattern map of CpG dyads. The bars sum up the DNA methylation status of all CpG dyads. The map next to the bar represents the distribution of methylated sites. Each column shows neighboured CpG dyads, and each line represents one sequence read. The reads in the map are sorted first by fully methylated sites and then by hemi-mCpG dyads. Red - fully methylated CpG dyads, light green and dark green - hemi-mCpG dyads on the upper and lower strand, blue - unmethylated CpG dyads, white - mutated or not analysable. We only observe influence of the Suv39h DKO on the DNA methylation of major Satellites. B: Non-CpG methylation. We could not detect changed non-CpG methylation pattern in the Suv39h DKO cells lines. (TIF) [file pgen.1002750.s006.tif]

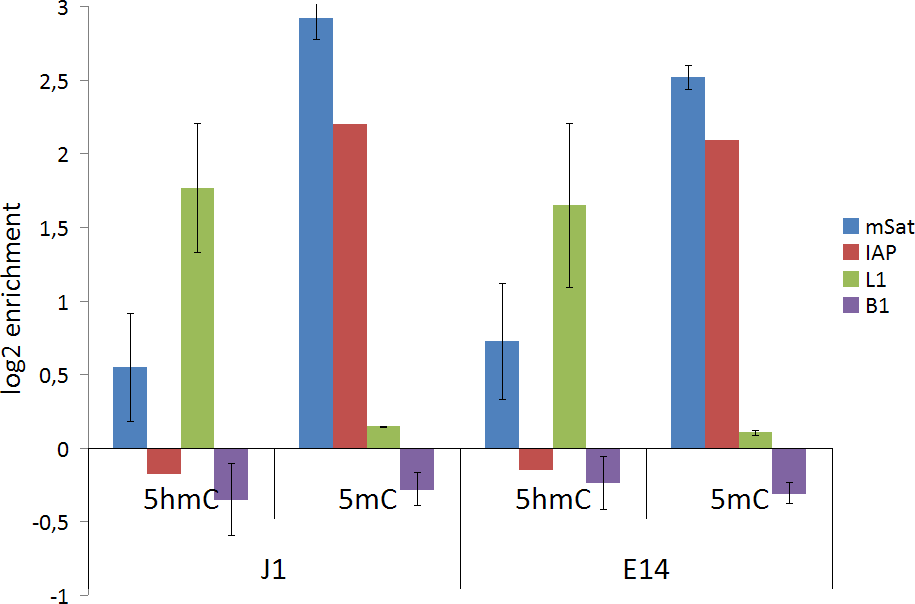

Supplement: Figure S7 — Relative enrichment of 5hmC and 5mC in the four analyzed repetitive elements. The raw data was taken from Ficz et al. [40]. Intriguing, L1 elements are highly enriched for 5hmC and only rarely for 5mC. IAPs are only enriched for 5mC. (TIF) [file pgen.1002750.s007.tif]

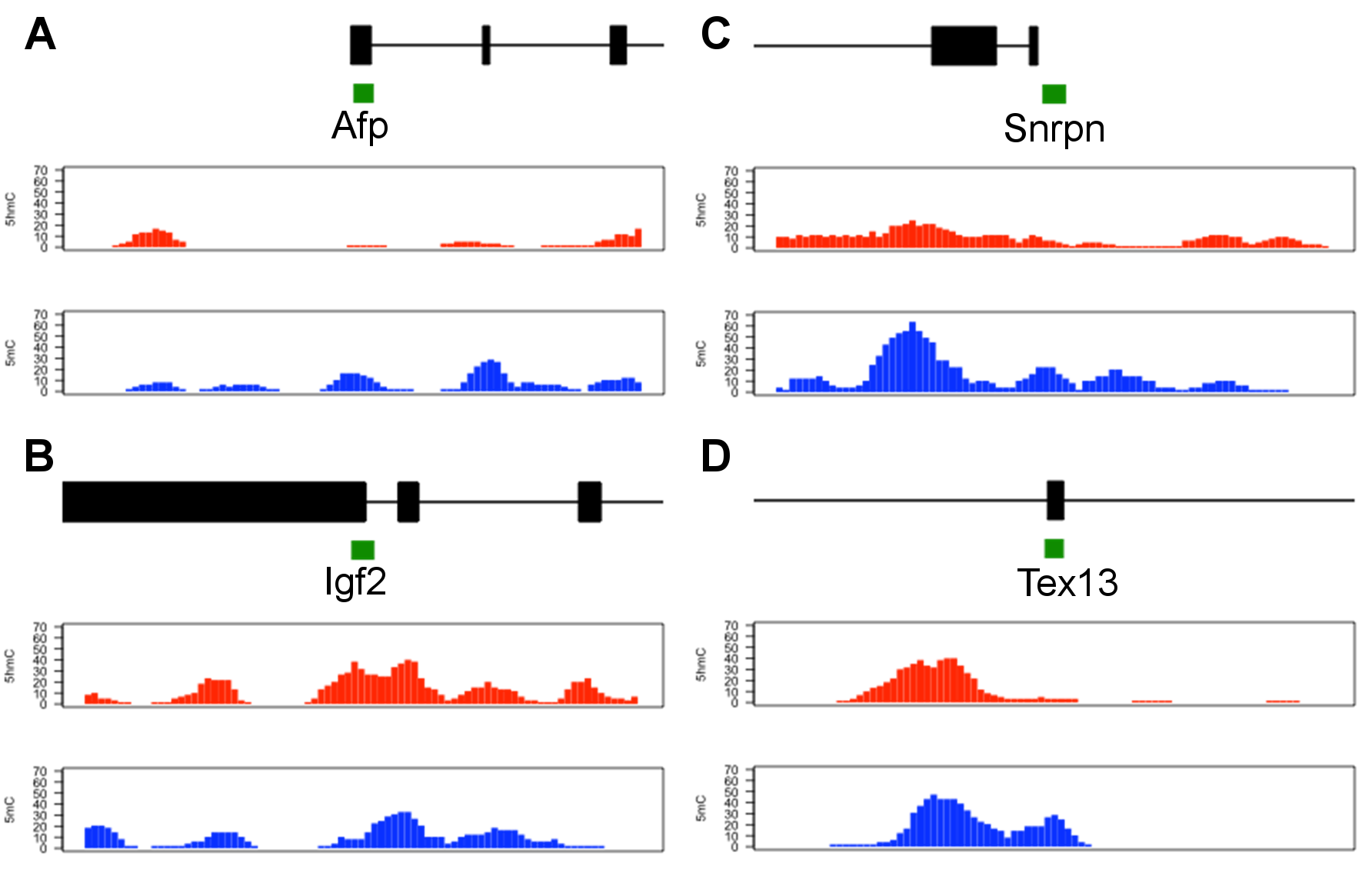

Supplement: Figure S8 — Enrichment profile of 5mDIP and 5hmDIP of Afp (A), Igf2 (B), Snrpn (C) and Tex13 (D). The raw data was taken from Ficz et al. [40]. Red shows the enrichment of 5hmC and blue the enrichment of 5mC. In green the position of the hairpin-bisulfite product is given. Only Igf2 shows enrichment of 5hmC in the analysed region. (TIF) [file pgen.1002750.s008.tif]

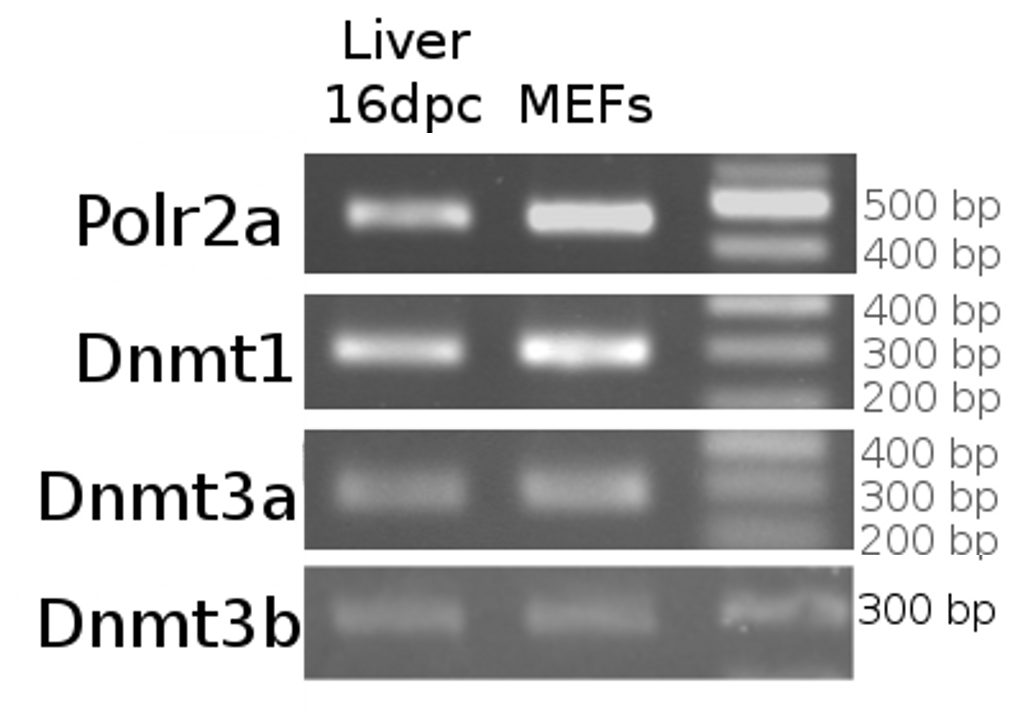

Supplement: Figure S9 — RT PCR for embryonic Liver and MEFs. The analysis shows that all three Dnmt transcripts are abundant in embryonic liver and cultivated MEFs. (TIF) [file pgen.1002750.s009.tif]

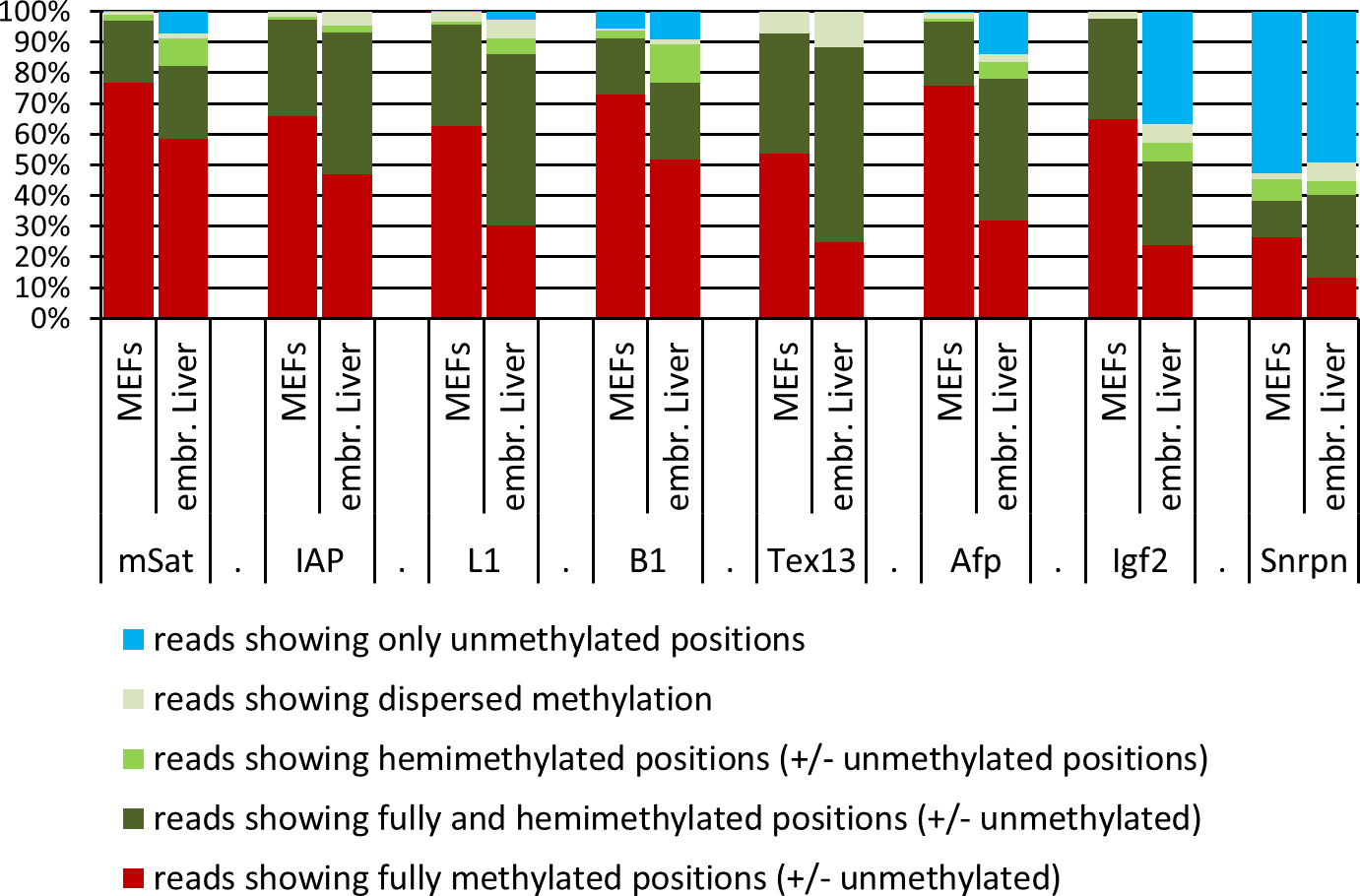

Supplement: Figure S10 — Occurrence of hemimethylated CpG positions in MEFs and embryonic liver. Given is the relative abundance of: reads showing either fully methylated positions (including or without unmethylated positions ( = +/−unmethylated positions)) in red, reads showing fully and hemimethylated positions (+/−unmethylated positions) in dark green, reads showing hemimethylated position (+/−unmethylated position) in green, reads showing dispersed methylation (hemimethylated sites on both strand) in light green and reads showing only unmethylated sites in blue. In embryonic liver, mainly reads showing fully and hemimethylated sites increase compared to MEFs. (TIF) [file pgen.1002750.s010.tif]

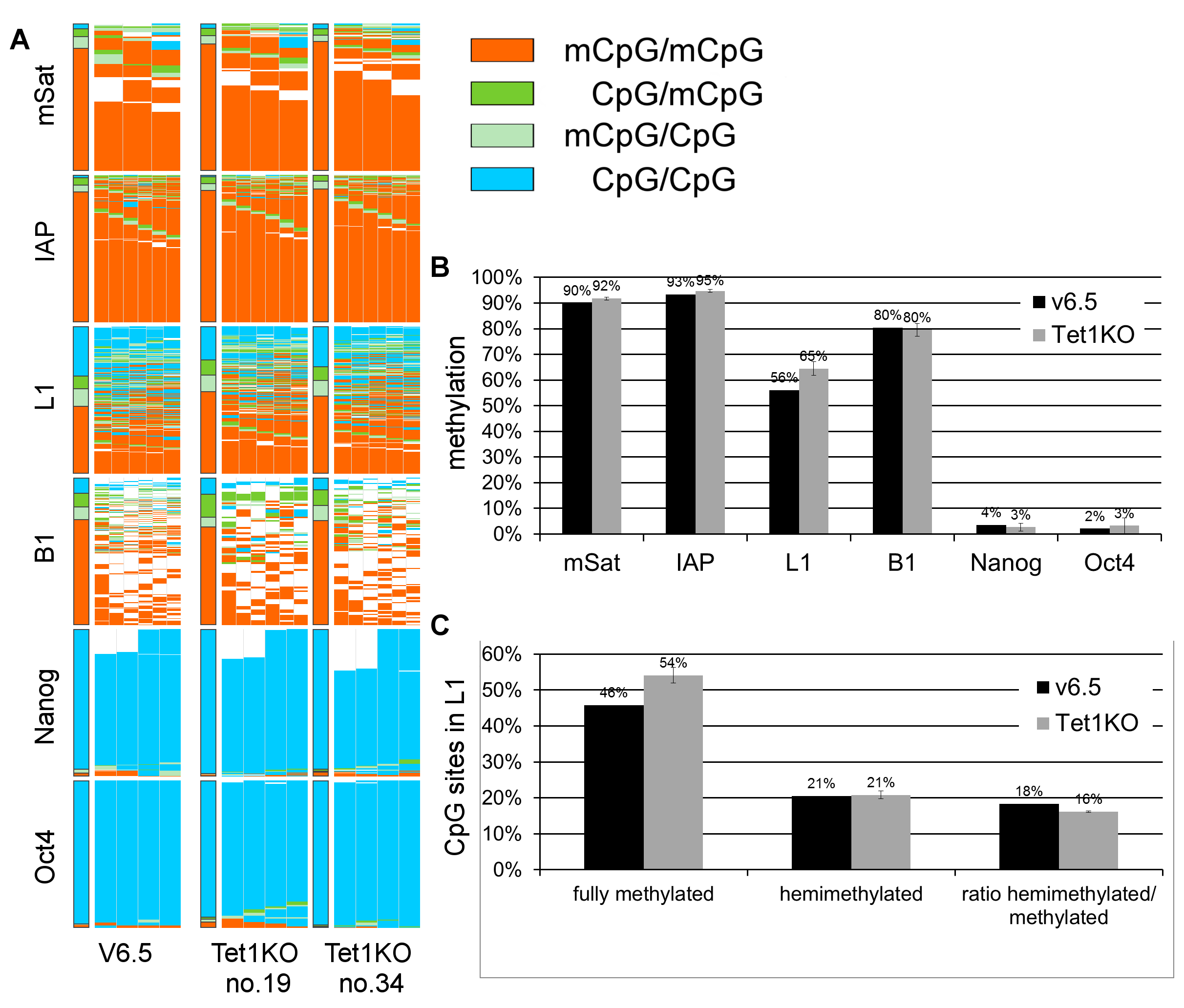

Supplement: Figure S11 — CpG methylation pattern map of repetitive elements in Tet1 KO ESCs. A+B: Only in L1 minor increase in methylation can be observed in the methylation pattern map (A, description see Figure S6a)) and the overall methylation level (B). Nanog and Oct4 promotor regions are unmethylated in WT and Tet1 KO ESCs. C: The increase in methylation goes along with increased amount of fully methylated sites. The amount of hemimethylated CpG dyads stays the same and relative to total methylation decreases slightly. (TIF) [file pgen.1002750.s011.tif]
