# Supplementary material for: In Vivo Control of CpG and Non-CpG DNA Methylation by DNA Methyltransferases
Source: PLoS Genet. 2012 Jun 28;8(6):e1002750. doi: 10.1371/journal.pgen.1002750 (PMC3386304; doi:10.1371/journal.pgen.1002750)
Supplement: Materials and Methods S1 — Information on the analyzed Cell-lines and detailed description of the hairpin-bisulfite analysis and the hidden Markov model. (PDF) [file pgen.1002750.s012.pdf]

Materials and Methods S1

Celllines and DNA

The Dnmt1-/- (Passage 10-15) J1 ESCs were homozygote for the c-allele (1). Dnmt3a (Passage 11), 3b (Passage 8) and 3a/3b (Passage 8) KO ESCs are described in Okano et al. (2). The Dnmt3L KO (Passage 15) is decribed in Hata et al. (3). Details on the Np95 E14 ESC KO are given in Sharif et al. (4) and details to the Suv39dn ESC line (Passage 46) and MEFs (Passage 25) in Lehnertz et al. (5). Embryonic liver DNA was obtained from a 16 dpc C57Bl/6 embryo. For the single copy genes passage number of the analysed ESCs are for Dnmt1 KO passage 17-22 and for Dnmt3a, Dnmt3b and Dnmt 3a/3b DKO passage 13-18.

Hairpinbisulfite Analysis

200 ng to 1 μg of DNA was restricted for 3 h with DdeI (NEB) for IAP, Eco47I (Fermentas) for mSat, MspI (NEB) for B1 and Igf2, NlaIII (NEB) for Snrpn, TaqI (NEB) for Afp, PstI (Fermentas) for Tex13 or 16 h with AccIII (Promega) for L1 , respectively. Ligation of the hairpinlinker (IAP: P-TTACCCGGTATATAGTATACCGGG, mSat: P-GACGGGCCTAATATAGTATAGGCCC, L1: P-CCGGGGGCCTATATAGTATAGGC-CC and B1+Igf2+Afp: P-CGTGGCCTAATATAGTATAGGCCA, Tex13: P-GGGCCT-AATATAGTATAGGCCCTGCA, Snrpn: P-GGGCCTAATATAGTATAGGCCCCATG) follows after precipitation or directly in the restriction buffer with 400 U of T4 Ligase (NEB). The Ligation Mix was bisulfite treated for 4 h at 50 °C including a first denaturing step for 15 min at 99 °C and two 5 min denaturing steps at 99 °C after 1 h and 2.5 h in 1.7 M sodium disulfite, 0.25 M NaOH both solved in H2O and 0.4 M 6-Hydroxy-2,5,7,8-tetramethylchroman-2-carbonacid (Sigma-Aldrich) solved in 1,4 Dioxan. Washing (1x TE) and desulfonation (10 min incubation in 0.3 M NaOH) steps followed on a microcon YM-30 column (Milipore, Schwalbach, Germany). Bisulfite DNA was eluted in 50 μl TE. PCR products were obtained in 35 cycles (for mSat, IAP, L1 and B1) or 40 cycles (for Afp, Tex13, Igf2 and Snrpn) from 2 μl of bisulfite product after 15 min at 97 °C for activation of HotFirePol (SolisBioDyne). For single copy genes HotStart-IT Binding Protein (Affymetrix) was added to the PCR reaction. Following primers (and conditions) were used for amplification of the specific elements: B1 5'TATTATGTAGTTTTGGTTGGTTTGGA3' and 5'TTTAAAACAAAATCTCAC-TATATAACCCT3' (95 °C 1 min, 55 °C 2 min, 72 °C 1 min), for IAP 5'TAAGAGTG-TAAGAAGTAAGAGAGAGAG3' and 5'ACAAAACTTTATTACTTACATCTTCAA3' (95 °C 1 min, 62 °C 45 sec, 72 °C 2 min), for Line1 5'TGGTAGTTTTTAGGTGGTATA-GAT3' and 5'TCAAACACTATATTACTTTAACAATTCCCA3' (95 °C 1 min, 55 °C 45 sec, 72 °C 90sec), for mSat 5'GGAAAATTTAGAAATGTTTAATGTAG3' and 5'AA-CAAAAAAACTAAAA ATCATAAAAA3' (95 °C 1 min, 56 °C 2 min, 72 °C 1 min). for Afp 5'TTTTGTTATAGGAAAATATTTTAAGTTA3' and 5'AAATCACAAAACATCT-TACCTATCC3' (95 °C 1 min, 57 °C 1min, 72 °C 1 min) for Tex13 5'AATAGTTGTTTTT-TATTTTTTGTTTGT3' and 5'CTCATTTTTTATTTACTTTTTTTTCTAAT3' (95 °C 1 min, 57 °C 1 min, 72 °C 1 min) for Igf2 5'GATATTTAGATGGGAGTTTAGGT-

TAAT3' and 5'CTACTAACTAACACCTCCTCTCCAA3' (95 °C 1 min, 57 °C 1 min, 72 °C 1 min), and for Snrpn 5'AGAATTTATAAGTTTAGTTGATTTTTT3' and 5'TAA-TCAAATAAAATACACTTTCACTACT3' (95 °C 1 min, 57 °C 1 min, 72 °C 1 min). At least two PCRs for each sample were mixed, purified and sequenced with standard 454 sequencing procedure. The methylation status of single CpG positions and non CpG-positions (after rermoval of CpGs in the reference and in the reads) was obtained with BiQAnalyzerHT (6). To obtain the methylation pattern on complementary CpG positions, the BiQ results where further processed using python scripts, including the removal of all reads showing a conversion rate in the linker which is less than 80%.

Estimation of methylation efficiencies using hidden Markov models

We model the state of a CpG dyad over time as a Markov chain $(X(t), t \in \mathbb{N})$ that takes values in $\{n, l, u, f\}$, i.e. the dyad is fully methylated (f), not methylated on both sides (n), methylated only at the lower strand (l), or upper strand (u). One time step of the chain corresponds to the time between two cell divisions, during which methylation is lost due to DNA replication and methylation is added due to the activity of Dnmts. We arrange the transition probabilities $\mathcal{P}(X(t+1) = y \mid X(t) = x)$ in the matrix P for all $x, y \in \{n, l, u, f\}$. If S is the matrix that describes the effect of DNA replication and M_1, M_{3a}, M_{3b} describe the effects of the methyltransferases Dnmt1, Dnmt3a, Dnmt3b, respectively, then we define the matrix product $P = SM_1M_{3a}M_{3b}$. Different orders of the matrices M_1, M_{3a}, M_{3b} are possible, but yield very similar results. In Table M1 and M2 we list the entries of the matrices S and M_1, where the row corresponds to the state at time t and the column to the state at time $t+1$. The entries of M_{3a} and M_{3b} are as for M_1 except that the index 1 is replaced by $3a$ and $3b$, respectively. E.g. the entry of M_{3a} that corresponds to the pair $(x, y) = (l, f)$ equals ρ_{3a}. The entries of S reflect the fact that DNA replication removes methylation with a probability of 0.5, while for M_1 we assume that with probability μ_1 de novo methylation occurs due to Dnmt1 and maintenance with probability ρ_1. Thus, if $X(t) \in \{l, u\}$ and no methylation is removed during DNA replication, then with probability ρ_1 the dyad is fully methylated due to Dnmt1. Let $\pi(0)$ be the row vector that contains the initial probability distribution of X.

	n	l	u	f
n	1	0	0	0
l	0.5	0.5	0	0
u	0.5	0	0.5	0
f	0	0.5	0.5	0

Table M1. Matrix S.

Then, after t cell generations, the distribution is $\pi(t) = \pi(0)P^t$. For each KO experiment, we assume that the initial methylation status corresponds to the equilibrium pattern of the WT, i.e., to the limiting distribution $\pi = \lim_{t\to\infty} \pi(t)$ of the Markov chain X that represents the methylation dynamics in WT. Thus, if $\tilde{\pi}_1(t)$ is the probability distribution

	n	l	u	f
n	1-μ_1	0.5μ_1	0.5μ_1	0
l	0	1-ρ_1	0	ρ_1
u	0	0	1-ρ_1	ρ_1
f	0	0	0	1

Table M2. Matrix M_1.

of the Markov chain that represents Dmnt1 KO, then $\tilde{\pi}_1(0) = \pi$ and $\tilde{\pi}_1(t) = \tilde{\pi}_1(0)\tilde{P}_1^t$, where $\tilde{P}_1 = SM_{3a}M_{3b}$. The distributions of the other KO experiments are defined in the same way.

We incorporated measurement errors as follows. If c is the rate of bisulfite conversion and $O(t)$ is the methylation pattern observed at time t, then the probabilities of $O(t)$ conditioned on the true methylation pattern $X(t)$ are

$$\begin{aligned}
\mathcal{P}(O(t) = n \mid X(t) = n) &= c^2 \\
\mathcal{P}(O(t) = u \mid X(t) = n) &= \mathcal{P}(O(t) = l \mid X(t) = n) = c(1-c) \\
\mathcal{P}(O(t) = f \mid X(t) = n) &= (1-c)^2 \\
\mathcal{P}(O(t) = u \mid X(t) = u) &= \mathcal{P}(O(t) = l \mid X(t) = l) = c \\
\mathcal{P}(O(t) = f \mid X(t) = u) &= \mathcal{P}(O(t) = f \mid X(t) = l) = 1-c \\
\mathcal{P}(O(t) = f \mid X(t) = f) &= 1
\end{aligned}$$

We assume that all other combinations have probability zero. The conversion rate of every sample was calculated from the unmethylated hairpinlinker (see Supplementary Table S2). Let C be the matrix with entries $\mathcal{P}(O(t) = y \mid X(t) = x)$. Then the probabilities $\mathcal{P}(O(t) = y)$ of the methylation pattern $y \in \{n, l, u, f\}$ after t cell generations in Dnmt1 KO are given by the vector $\tilde{\pi}_1(t)C = \tilde{\pi}_1(0)\tilde{P}_1^tC$. Note that both $\tilde{\pi}_1(t)$ and C depend on the chosen element, e.g., on IAP, mSat, Line1, or B1 among others. Now, assuming independence of the observations, we compute the likelihood of observing k_n, k_l, k_u, k_f samples of the four patterns n, l, u, f as

$$\mathcal{L} = \mathcal{P}(O(t) = n)^{k_n} \cdot \mathcal{P}(O(t) = l)^{k_l} \cdot \mathcal{P}(O(t) = u)^{k_u} \cdot \mathcal{P}(O(t) = f)^{k_f}.$$

We evaluated the likelihood after the number t of cell generations when the hairpin bisulfite conversion was performed. This cell generation number of the analysed cell lines was estimated by multiplying the passage number with 3.25, which for example yields $t = 41$ for Dnmt1 KO, $t = 36$ for Dnmt3a KO, $t = 26$ for Dnmt3b, and $t = 26$ for Dnmt3a/b DKO in the case of mSat. Note that the likelihood $\mathcal{L}$ depends on the matrix $\tilde{P}_1$ and therefore on the methylation efficiencies μ_{3a}, μ_{3b}, ρ_{3a}, ρ_{3b} (since Dnmt1 is KO, the likelihood does not depend on the parameters μ_1, ρ_1). We then maximize the likelihood w.r.t. μ_{3a}, μ_{3b}, ρ_{3a}, ρ_{3b} by using MATLAB's Global Search, which is a global optimization method for non-linear problems.

The likelihoods of the remaining KO experiments, i.e., Dnmt3a KO, Dnmt3b KO, and Dnmt3a/b KO, are defined in the same way and depend on the methylation efficiencies of the respective involved Dnmts.

During the computation of the estimated probabilities, the standard deviations of the estimators have been derived from the corresponding Fisher information matrices.

Transcription analysis of Dnmts

Total RNA was isolated from cultured MEFs and embryonic liver 16 dpc using Qiagen AllPrep Kit. The RNA was analysed for Polr2a, Dnmt1, Dnmt3a and Dnmt3b transcripts by RT-PCR using following primers: Polr2a 5'ACCAAAGAGAAGGGCCATGGCG3' and 5'TTCTGCATGGCACGGGGTAAGC3', Dnmt1 5'CATGGTGCTGAAGCTCACACTGC3' and 5'GGGAGGTCAGACATGGTGTCTCG3', Dnmt3a 5'T GAGTTCTACCGCCTCCTGC3' and 5'ATTCTGCCGTGCTCCAGACA3', Dnmt3b 5' TGGGTACAGTGGTTTGGTGATGGCA3' and 5'TGGTTGCTTCTTGTTGGGTTTGAGG3'.

References

1. Lei, H., et al. (1996) De novo DNA cytosine methyltransferase activities in mouse embryonic stem cells. *Development* 122: 3195-3205.

2. Okano, M., Bell, D. W., Haber, D. A.Li, E. (1999) DNA methyltransferases Dnmt3a and Dnmt3b are essential for de novo methylation and mammalian development. *Cel*l 99: 247-257.

3. Hata, K., Okano, M., Lei, H.Li, E. (2002) Dnmt3L cooperates with the Dnmt3 family of de novo DNA methyltransferases to establish maternal imprints in mice. *Development* 129: 1983-1993.

4. Sharif, J., et al. (2007) The SRA protein Np95 mediates epigenetic inheritance by recruiting Dnmt1 to methylated DNA. *Nature* 450: 908-912.

5. Lehnertz, B., et al. (2003) Suv39h-mediated histone H3 lysine 9 methylation directs DNA methylation to major satellite repeats at pericentric heterochromatin. *Curr Biol* 13: 1192-1200.

6. Lutsik, P., et al. (2011) BiQ Analyzer HT: locus-specific analysis of DNA methylation by high-throughput bisulfite sequencing. *Nucleic Acids Res* 39 Suppl 2: W551-556.
