# Supplementary material for: In Vivo Control of CpG and Non-CpG DNA Methylation by DNA Methyltransferases
Source: PLoS Genet. 2012 Jun 28;8(6):e1002750. doi: 10.1371/journal.pgen.1002750 (PMC3386304; doi:10.1371/journal.pgen.1002750)
Supplement: Table S1 — Used reference sequences for the hairpin-bisulfite analysis of the repetitive elements. (DOCX) [file pgen.1002750.s013.docx]

| **mSat** | ggaaaatttagaaatgtccaatgtaggacgtggaatatggcaagaaaactgaaaatcat |
| --- | --- |
|  | gggaaatgagaaacatccacttgtcgacttgaaaaatgacgaaatcactaaaaaacgtg |
|  | aaaaatgagaaatgcacactgaaggacc |
| **IAP** | ctaagtggtaaacaaataatctgcgcatatgccgagggtggttctctactccatgtgct |
|  | ctgccttccccgtgacgtcaactcggccgatgggctgcagccaatcagggagtgacacg |
|  | tcctaggcgaaatataactctcctaaaaaagggacggggtttcgttttctctctctctt |
|  | gcttcttacactcttgctcctgaagatgtaagcaataaagttttgc |
| **B1** | cgaggcctaatatagtataggcctcgggcgtggtggcgcacgcctttaatcccagcact |
|  | cgggaggcagaggcaggcggatctctgtgagttcgaggccagcctggtctacatagcga |
|  | gttccaggccagccagggctacatagtgagaccctgtctcaaa |
| **L1** | tccggaccggaggacaggtgcccacccggctggggaggcggcctaagccacagcagcag |
|  | cggtcgccatcttggtcccgggactccaaggaacttaggaatttagtctgcttaagtga |
|  | gagtctgtaccacctgggaactgccaaagcaacacagtgtctgagaaaggtcctgtttt |
|  | gg |
