# Supplementary material for: In Vivo Control of CpG and Non-CpG DNA Methylation by DNA Methyltransferases
Source: PLoS Genet. 2012 Jun 28;8(6):e1002750. doi: 10.1371/journal.pgen.1002750 (PMC3386304; doi:10.1371/journal.pgen.1002750)
Supplement: Table S2 — Number of reads, analysed CpG positions and conversion rates of the linker sequences (the conversion rate in each sample was calculated from the linker sequence, which contains 5 to 7 unmethylated Cs) A For analyzed repetitive elements and B For analyzed single copy genes. (DOCX) [file pgen.1002750.s014.docx]

**A**

|  | **major Satellites** | | | **IAPLTR1** | | | **L1Md_Gf 5'UTR** | | | **B1** | | |
| --- | --- | --- | --- | --- | --- | --- | --- | --- | --- | --- | --- | --- |
|  | reads | CpGs | conv. | reads | CpGs | conv. | reads | CpGs | conv. | reads | CpGs | conv. |
|  |  |  | (%) |  |  | (%) |  |  | (%) |  |  | (%) |
| ESC J1 | 3141 | 8511 | 99.8 | 722 | 4176 | 99.6 | 2097 | 12307 | 99.6 | 412 | 1213 | 98.0 |
| ESC Dnmt1 KO | 1729 | 4596 | 99.7 | 863 | 4560 | 99.3 | 1188 | 7062 | 99.5 | 303 | 875 | 98.1 |
| ESC Dnmt3a KO | 893 | 2338 | 99.5 | 524 | 3048 | 99.7 | 831 | 4901 | 99.4 | 146 | 396 | 98.9 |
| ESC Dnmt3b KO | 1316 | 3537 | 99.6 | 803 | 4633 | 99.4 | 1182 | 6974 | 99.5 | 303 | 919 | 97.9 |
| ESC Dnmt3a/b DKO | 1643 | 4438 | 99.8 | 648 | 3568 | 99.5 | 975 | 5699 | 99.5 | 322 | 958 | 98.3 |
| ESC Dnmt3L KO | 1106 | 2880 | 99.5 | 381 | 2183 | 99.5 | 1185 | 6896 | 99.2 | 618 | 1784 | 99.5 |
| ESC E14 | 1060 | 2791 | 99.5 | 457 | 2604 | 99.7 | 838 | 4829 | 99.4 | 863 | 2459 | 99.1 |
| ESC Np95 KO | 1471 | 3892 | 99.6 | 336 | 1932 | 99.3 | 639 | 3652 | 99.3 | 547 | 1576 | 99.1 |
| ESC WT26 | 469 | 1246 | 99.6 | 396 | 2288 | 99.7 | 752 | 4387 | 99.4 | 161 | 461 | 99.2 |
| ESC Suv39h KO | 567 | 1522 | 99.6 | 415 | 2371 | 99.6 | 765 | 4341 | 99.5 | 318 | 863 | 98.9 |
| MEFs W8 | 728 | 1900 | 99.6 | 313 | 1769 | 99.7 | 840 | 4623 | 99.2 | 410 | 1217 | 99.4 |
| MEFs Suv39h KO | 595 | 1554 | 99.6 | 708 | 4083 | 99.9 | 547 | 2969 | 99.4 | 469 | 1341 | 99.4 |
| Liver 16dpc | 661 | 1734 | 99.4 | 481 | 2820 | 99.6 | 474 | 2736 | 99.2 | 374 | 1011 | 99.2 |

**B**

|  | **Tex13** | | | **Afp** | | | **Igf2** | | | **Snrpn** | | |
| --- | --- | --- | --- | --- | --- | --- | --- | --- | --- | --- | --- | --- |
|  | reads | CpGs | conv. | reads | CpGs | conv. | reads | CpGs | conv. | reads | CpGs | conv. |
|  |  |  | (%) |  |  | (%) |  |  | (%) |  |  | (%) |
| ESC J1 | 1199 | 11775 | 99.2 | 625 | 3014 | 99.5 | 483 | 3783 | 98.9 | 326 | 3217 | 98.8 |
| ESC Dnmt1 KO | 481 | 4699 | 99.4 | 153 | 734 | 99.7 | 281 | 2152 | 99 | 167 | 1631 | 99.8 |
| ESC Dnmt3a KO | 528 | 5179 | 99.6 | 498 | 2444 | 99 | 463 | 3597 | 99 | 324 | 3213 | 99.5 |
| ESC Dnmt3b KO | 916 | 8990 | 99.3 | 400 | 1933 | 99.3 | 394 | 3048 | 99 | 237 | 2340 | 99.3 |
| ESC Dnmt3a/b DKO | 714 | 6989 | 99.6 | 216 | 1024 | 99.1 | 315 | 2394 | 99 | 241 | 2365 | 99.7 |
| ESC Dnmt3L KO | 604 | 5892 | 99.4 | 294 | 1435 | 99.1 | 321 | 2496 | 99.3 | 192 | 1887 | 99.4 |
| MEFs W8 | 1183 | 11654 | 99.3 | 205 | 900 | 99.7 | 254 | 1906 | 99.3 | 251 | 2433 | 99.6 |
| Liver 16dpc | 743 | 7263 | 99.6 | 222 | 1012 | 99.6 | 138 | 1047 | 98.7 | 329 | 3159 | 99.6 |
