# Supplementary material for: In Vivo Control of CpG and Non-CpG DNA Methylation by DNA Methyltransferases
Source: PLoS Genet. 2012 Jun 28;8(6):e1002750. doi: 10.1371/journal.pgen.1002750 (PMC3386304; doi:10.1371/journal.pgen.1002750)
Supplement: Table S3 — De novo and maintenance methylation efficiencies of Dnmts. Fitted efficiency values with standard deviations using maximum likelihood method, assuming that de novo methylation has the same probability to methylate unmethylated or hemimethylated positions. The values give the probabilities of Dnmt1, Dnmt3a and Dnmt3b mediated de novo or maintenance methylation per replication. (DOCX) [file pgen.1002750.s015.docx]

|  |  | **Dnmt1** | | **Dnmt3a** | | **Dnmt3b** | |
| --- | --- | --- | --- | --- | --- | --- | --- |
|  |  | Hemi-methylated | *Un-methylated* | Hemi-methylated | *Un-methylated* | Hemi-methylated | *Un-methylated* |
| **mSat** | **efficiency** | 0.901 | 0.321 | 0.333 | 0.203 | 0.134 | 0.104 |
|  | **STDEV** | 0.003 | 0.010 | 0.017 | 0.017 | 0.014 | 0.019 |
| **IAP** | **efficiency** | 0.933 | 0.368 | 0.258 | 0.132 | 0.238 | 0.069 |
|  | **STDEV** | 0.002 | 0.013 | 0.022 | 0.020 | 0.015 | 0.021 |
| **Tex13** | **efficiency** | 0.945 | 0.115 | 0.000 | 0.183 | 0.322 | 0.077 |
|  | **STDEV** | 0.003 | 0.012 | 0.029 | 0.009 | 0.013 | 0.009 |
| **Afp** | **efficiency** | 0.947 | 0.055 | 0.294 | 0.258 | 0.000 | 0.305 |
|  | **STDEV** | 0.003 | 0.010 | 0.585 | 0.102 | 0.765 | 0.221 |
| **L1** | **efficiency** | 0.746 | 0.019 | 0.258 | 0.142 | 0.000 | 0.034 |
|  | **STDEV** | 0.041 | 0.031 | 0.975 | 0.045 | 0.982 | 0.073 |
| **B1** | **efficiency** | 0.897 | 0.010 | 0.238 | 0.286 | 0.000 | 0.100 |
|  | **STDEV** | 0.025 | 0.006 | 1.126 | 0.015 | 1.095 | 0.270 |
| **Igf2** | **efficiency** | 0.798 | 0.009 | 0.100 | 0.123 | 0.112 | 0.071 |
|  | **STDEV** | 0.006 | 0.001 | 0.039 | 0.004 | 0.022 | 0.004 |
