# Supplementary material for: In Vivo Control of CpG and Non-CpG DNA Methylation by DNA Methyltransferases
Source: PLoS Genet. 2012 Jun 28;8(6):e1002750. doi: 10.1371/journal.pgen.1002750 (PMC3386304; doi:10.1371/journal.pgen.1002750)
Supplement: Table S4 — Prediction of WT methylation. Shown are the predictions for the methylation levels of the repetitive elements in WT J1 ESCs using all fitted parameters (predicted). As reference the experimental derived methylation level of WT J1 ESCs is listed (data). (DOCX) [file pgen.1002750.s016.docx]

|  |  | **CpG/CpG** | **mCpG/CpG + CpG/mCpG** | **mCpG/mCpG** |
| --- | --- | --- | --- | --- |
| **mSat** | **predicted** | 0.038 | 0.081 | 0.880 |
|  | **data** | 0.021 | 0.094 | 0.885 |
| **IAP** | **predicted** | 0.028 | 0.053 | 0.920 |
|  | **data** | 0.007 | 0.094 | 0.899 |
| **Tex13** | **predicted** | 0.046 | 0.054 | 0.892 |
|  | **data** | 0.024 | 0.046 | 0.930 |
| **Afp** | **predicted** | 0.032 | 0.068 | 0.900 |
|  | **data** | 0.005 | 0.070 | 0.925 |
| **L1** | **predicted** | 0.402 | 0.185 | 0.413 |
|  | **data** | 0.368 | 0.226 | 0.406 |
| **B1** | **predicted** | 0.113 | 0.128 | 0.759 |
|  | **data** | 0.054 | 0.159 | 0.787 |
| **Igf2** | **predicted** | 0.353 | 0.169 | 0.479 |
|  | **data** | 0.292 | 0.159 | 0.549 |
